# Discovery of Cysteine and Its Derivatives as Novel Antiviral and Antifungal Agents

**DOI:** 10.3390/molecules26020383

**Published:** 2021-01-13

**Authors:** Shan Yang, Tienan Wang, Yanan Zhou, Li Shi, Aidang Lu, Ziwen Wang

**Affiliations:** 1 School of Chemical Engineering and Technology, Hebei University of Technology, Tianjin 300130, China; 17865513875@163.com (S.Y.); cdwangtienan@163.com (T.W.); zyn990611@163.com (Y.Z.); s18310724767@163.com (L.S.); 2 Tianjin Key Laboratory of Structure and Performance for Functional Molecules, College of Chemistry, Tianjin Normal University, Tianjin 300387, China

**Keywords:** natural product, cysteine and its derivatives, anti-TMV activity, antifungal activity, mode of action, molecular docking

## Abstract

Based on the structure of the natural product cysteine, a series of thiazolidine-4-carboxylic acids were designed and synthesized. All target compounds bearing thiazolidine-4-carboxylic acid were characterized by ^1^H-NMR, ^13^C-NMR, and HRMS techniques. The antiviral and antifungal activities of cysteine and its derivatives were evaluated in vitro and in vivo. The results of anti-TMV activity revealed that all compounds exhibited moderate to excellent activities against tobacco mosaic virus (TMV) at the concentration of 500 μg/mL. The compounds cysteine (**1**), **3**–**4**, **7**, **10**, **13**, **20,** **23**, and **24** displayed higher anti-TMV activities than the commercial plant virucide ribavirin (inhibitory rate: 40, 40, and 38% at 500 μg/mL for inactivation, curative, and protection activity in vivo, respectively), especially compound **3** (inhibitory rate: 51%, 47%, and 49% at 500 μg/mL for inactivation, curative, and protection activity in vivo, respectively) with excellent antiviral activity emerged as a new antiviral candidate. Antiviral mechanism research by TEM exhibited that compound **3** could inhibit virus assembly by aggregated the 20S protein disk. Molecular docking results revealed that compound **3** with higher antiviral activities than that of compound **24** did show stronger interaction with TMV CP. Further fungicidal activity tests against 14 kinds of phytopathogenic fungi revealed that these cysteine derivatives displayed broad-spectrum fungicidal activities. Compound **16** exhibited higher antifungal activities against *Cercospora arachidicola Hori* and *Alternaria solani* than commercial fungicides carbendazim and chlorothalonil, which emerged as a new candidate for fungicidal research.

## 1. Introduction

Fungal and viral pathogens can induce various of plant diseases such as those causing brown spots on peanut leaves, ring rots on stem and fruit of apples, gray mold on cucumbers and grapes, leading to huge losses to agriculture and horticulture production and threatening food security [1]. Tobacco mosaic virus (TMV), known as “plant cancer”, not only can seriously harm tobacco, but also can infect potato, pepper, tomato, eggplant, nightshade, and more than 400 other plant species [2,3]. The long-term, large-scale use of traditional high-toxic pesticides not only forces bacteria and viruses to develop resistance, but also poses a threat to human health. The development of new antifungal and antiviral agents with unique mode of action is becoming more and more urgent for plant protection and agricultural production [4,5,6].

Amino acids are substances that exist widely in Nature. Cysteine is the only sulfur-containing amino acid with the ability to form disulfide bonds. Due to the bigger atomic radius of sulfur and the lower dissociation energy of the S−H bond, the thiol group of cysteine possesses the ability to perform both nucleophilic and redox-active functions which are unfeasible for the other natural amino acids [7,8,9]. Cysteine, with its easily modified molecular structure, has attracted attention from biological and chemical scientists. A series of biological activities of cysteine and its derivatives have been reported, such as cytotoxicity [10], neurotoxicity [11,12,13], oxidant activity [14], accelerated DNA oxidative damage [15], and so on.

Heterocycles are an important framework for the development of new drugs, especially S-containing heterocycles which have been found to have the ability to induce apoptosis of various cells [16]. Thiazolidine drugs containing N and S atoms can exert drug effects through various mechanisms of action, such as inhibiting neuraminidase of influenza A virus [17], inhibiting protein synthesis [18], accelerating cell apoptosis [16,19], enhancing antioxidant capacity [7], immune stimulation [20], etc. Some thiazolidine-4-carboxylic acid derivatives can also oxidatively cleave DNA and interact with metal ions [21]. Some thiazolidine derivatives have been used in a variety of synthetic modifications due to their simple structure, diverse biological characteristics, and excellent environmental compatibility [22,23].

**Figure 1:**
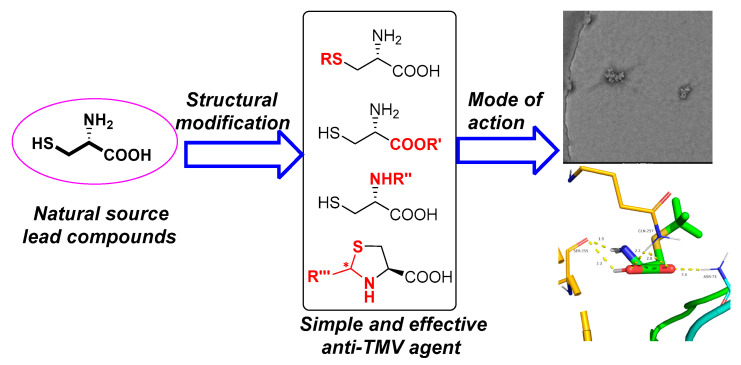
Design of cysteine derivatives.

It is an important research direction for us to find effective antiviral candidates based on natural products [24]. In our previous work, a series of amino acid gossypol Schiff bases were designed and synthesized. The structure-activity relationship revealed that both the carboxy group and substituents in amino acids had significant effect on anti-TMV [25]. Combined with our existing work experience and the above findings, we made systematic research on cysteine and its derivatives (Figure 1) in the present work. All synthetic compounds were characterized by ^1^H-NMR, ^13^C-NMR, and HRMS. The antiviral and antifungal activities of these compounds and the structure-activity relationship were evaluated systematically.

## 2. Results

### 2.1. Chemistry

**Figure 2:**
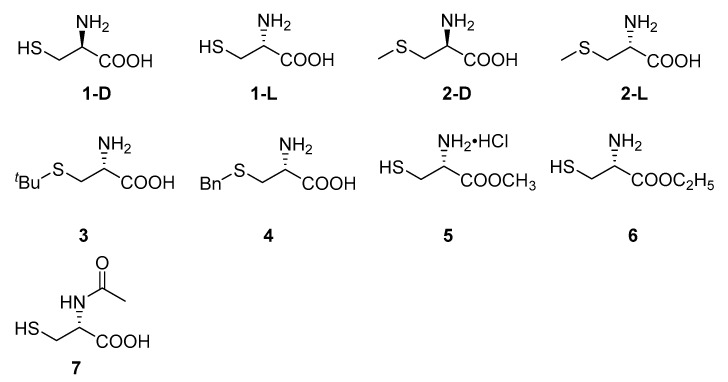
Structures of compounds **1**–**7.**

**Scheme 1:**
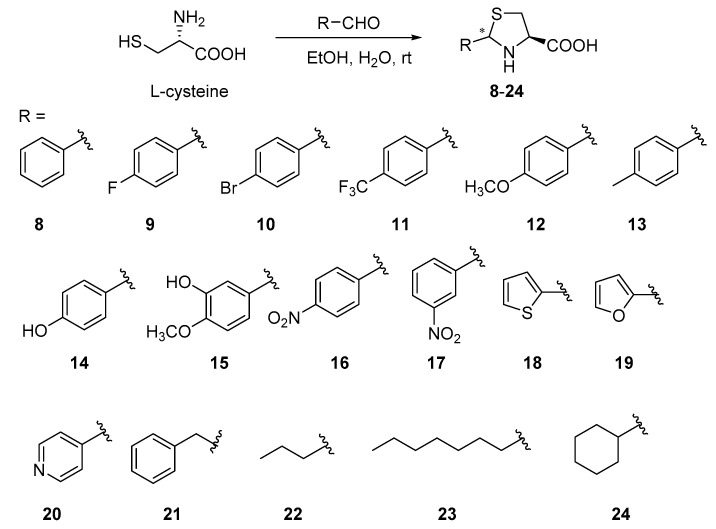
Synthesis of **8**–**24.**

Cysteine and its derivatives **1**–**7** (Figure 2) were purchased directly. A series of thiazole ring containing L-cysteine derivatives **8**–**24** were designed and synthesized from L-cysteine and different substituted aldehydes (Scheme 1) by a one-pot method [7,26].

### 2.2. Phytotoxic Activity

The phytotoxicity-activity tests revealed that cysteine and its derivatives were safe for testing on plants at 500 μg/mL. The detailed test procedures can be seen in our previous reports [5,27]. The detailed test procedures can also be found in the Supplementary Materials.

### 2.3. Antiviral Activity

#### 2.3.1. In Vitro Anti-TMV Activity

The anti-TMV activities of cysteine and its derivatives **1**–**24** are listed in Table 1 with the commercial drugs ribavirin and ningnanmycin as controls. Compounds **1**–**7** had better anti-TMV activities than ribavirin. Among compounds **1**–**7**, compounds **3** (inhibitory rate: 48% at 500 μg/mL) and **4** (inhibitory rate: 45% at 500 μg/mL) showed better anti-TMV activities than the others. At the concentration of 100 μg/mL, compounds **3** (inhibitory rate: 13%) and **4** (inhibitory rate: 12%) also displayed better anti-TMV activities than the commercial plant virucide ribavirin (inhibitory rate: 7%). Most of the cysteine derivatives **8**–**24** with thiazolidine structure exhibited better anti-TMV activities than that of the commercial plant virucide ribavirin. Especially, compounds **23** (inhibitory rate: 46% at 500 μg/mL) and **24** (inhibitory rate: 45% at 500 μg/mL) showed 10% higher anti-TMV activity than ribavirin (inhibitory rate: 35% at 500 μg/mL).

#### 2.3.2. In Vivo Anti-TMV Activity

In vivo anti-TMV activity includes three test modes: inactivation, curative, and protection. As shown in Table 2, most of the compounds also displayed higher in vivo activities than ribavirin. Compound **3** displayed the best anti-TMV activity at 500 μg/mL (inactivation activity, 51%; curative activity, 47%; protection activity, 49%), which is significantly higher than that of ribavirin (inactivation activity, 40%; curative activity, 40%; protection activity, 38%).

### 2.4. Mode of Action Studies

#### 2.4.1. Preliminary Mode of Action

Preliminary mode of action revealed that these compounds can inhibit the assembly of TMV. The detailed method was described in the literature [24] and also can be found in the Supporting Materials.

#### 2.4.2. Docking Studies

To further study the mechanism of the interaction between cysteines and TMV CP, we chose AutoDock Vina 1.1.2 for molecular docking [28]. The docking poses are ranked according to their docking sites and the lowest binding energy of macromolecule-ligand complex is considered being the best. It can be proved H-bond interaction and strong binding affinity between cysteines and TMV CP.

### 2.5. Fungicidal Activity

#### 2.5.1. In Vitro Fungicidal Activity

We further tested the inhibitory effects of compounds **1**–**24** on 14 common agricultural pathogens at a concentration of 50 μg/mL using fungicidal growth rate assay [27,29] with commercial fungicidal agents chlorothalonil and carbendazim as controls. All compounds showed broad-spectrum fungicidal activities (Table 3). Compound **16** exhibited higher antifungal activities against *Cercospora arachidicola Hori* (inhibitory rate: 71%) and *Alternaria solani* (inhibitory rate: 58%) than commercial fungicides carbendazim and chlorothalonil. Compound **16** had an inhibitory rate of 83% against *Physalospora piricola.*

#### 2.5.2. In Vivo Fungicidal Activity

Compounds **1**–**24** were further tested in vivo fungicidal activity at a concentration of 200 μg/mL using standard methods [29] with azoxystrobin as a control against *Blumeria graminis* f.sp. *tritici*, *Sclerotinia sclerotiorum*, *Botrytis cinereal*, *Rhizoctonia solani*, *Corynespora cassiicola*, and *Phytophthora capsica* 6 kinds of pathogenic fungi. As shown in Table 4, most of these compounds also displayed broad-spectrum in vivo fungicidal activities. Compounds **12** and **18** showed 20% inhibition rate against *Rhizoctonia solani*. Compound **12** exhibited higher activity than other compounds against *Botrytis cinereal*. The inhibition rate of compound **18** is more than 20% against *Corynespora cassiicola*.

## 3. Discussion

### 3.1. Synthesis

Compounds **8**–**24** bearing a thiazolidine ring based on L-cysteine were synthesized under basic conditions by a one-pot method in high to nearly quantitative yields. The cyclization of aldehydes with cysteine has been reported in the literature under conditions using a water/ethanol mixture (50:50, *v*:*v*) [7,26]. To promote the precipitation of the product from the reaction system and improve the yield of the reaction, the amount of ethanol was reduced based on references. The yields of compounds **8**–**24** ranged from 82% to 99%. Two nucleophilic attacks of the aldehyde produced the closed ring structure and led to the generation of a new uncontrolled chiral center. Thus, compounds **8**–**24** are obtained as diastereomeric mixtures [7]. Although the 2*R*, 4*R* and 2*S*,4*R* isomers were mixed, the distinctive singlet around 5.5 ppm of the hydrogen on C-2 gave a clearly distinguishable ratio of the isomers [7].

### 3.2. Phytotoxic Activity

Healthy growing 5–6 leaf stage tobaccos (*Nicotiana tabacum var Xanthi nc*) were slected for phytotoxic activity tests. The fresh solutions (500 μg/mL) of compounds **1**–**24** were gently smeared on the leaves. One hour after treatment, all the treated tobacco leaves were observed intact. The growth of tobacco leaves was continuously observed and calculated the weight after 3, 7, and 10 days, respectively. Encouragingly, none of the compounds were toxic to tobacco leaves.

### 3.3. Structure-Activity Relationship of the Antiviral Activity

As the results of Table 1, compounds **1**–**7** had better anti-TMV activities than ribavirin. Chirality has no obvious effect on the antiviral activities of these compounds (inhibitory effect: **1**-**D** ≈ **1**-**L**, **2-D** ≈ **2**-**L**). Therefore, in the follow-up study, we only used L-cysteine derivatives to explore the effect of different substituents. The antiviral activity was increased slightly when the S atom and N atom of cysteine had substituents (antiviral activity: **3** > **4** > **7** > **1**, **2**, **5**, **6**). Cyclization is an important method to improve molecular stability and biological activity. A series of thiazolidine-4-carboxylic acid-containing compounds **8**–**24** were designed and synthesized. As shown in Table 1, most of the designed compounds displayed better antiviral activities than ribavirin. For the substituted benzene compounds **8**–**17**, the electron-withdrawing group substitution at the para position of the benzene ring is beneficial to the improvement of biological activity (inhibitory effect: **11** > **16** > **10**, **17** > **9** > **8**). When introduction of electron-donating groups such as CH_3_O (**12**), CH_3_ (**13**) into the para position of the benzene ring, the anti-TMV activity is also improved (inhibitory effect: **13** > **12** > **15** > **8**, **17** > **9** > **8**). However, the activity is sharply declined after the introduction of -OH at the *para*-position of the benzene ring (**14**). When the 2-position of thiazolidine structure is heterocyclic groups, such as thiophene (**18**), furan (**19**), pyridine (**20**), only compound **20** displayed better anti-TMV activity. Compounds **21**–**24** with the aliphatic groups at 2-position of thiazolidine exhibited moderate to excellent anti-TMV activities, the introduction of long-chain fat groups (**23**), and cyclohexyl groups (**24**) at 2-position of thiazolidine can lead to an increase in activity (**23** > **24** > **35** > **22**).

Just like the antiviral activity in vitro, compounds **2**−**24** displayed moderate to good in vivo activities (Table 2), and the activities of compounds **3**, **4**, **7**, **13**, **23**, **24** are significantly higher than that of cysteine. In particular, compound **3** showed excellent activity against TMV (inhibitory rate: 51%, 47%, and 49% at 500 μg/mL for inactivation, curative, and protection activity in vivo, respectively). Compounds **4**, **7**, **10**, **13**, **20**, **23**, and **24** displayed higher anti-TMV activities than the commercial plant virucide ribavirin (inhibitory rate: 40, 40, and 38% at 500 μg/mL for inactivation, curative, and protection activity in vivo, respectively). Compounds **2**, **10**, **12**, **16** exhibited approximate anti-TMV activities as cysteine. The structure-activity relationship revealed that the substitutions of S atom and N atom had a great influence on the anti-TMV activity.

### 3.4. Study on the Mechanism of Anti-TMV Activity

#### 3.4.1. Preliminary Mode of Action

**Figure 3:**
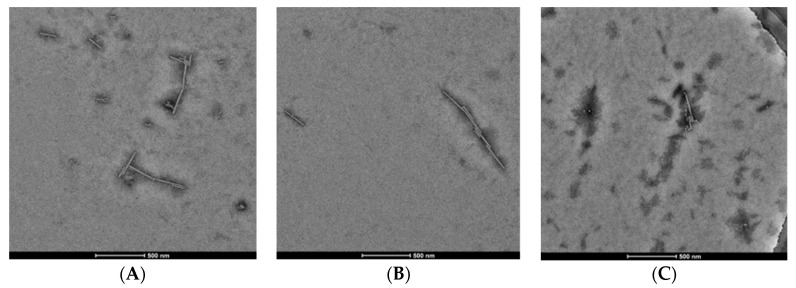
TMV rod assembly inhibition of compound **3** and NK0209: (**A**) 20S CP disk + RNA (500 nm scale bar), (**B**) 20S CP disk + RNA + 1/100 DMSO (500 nm scale bar), (**C**) 20S CP disk + RNA + 10 μM **3** (500 nm scale bar).

**Figure 4:**
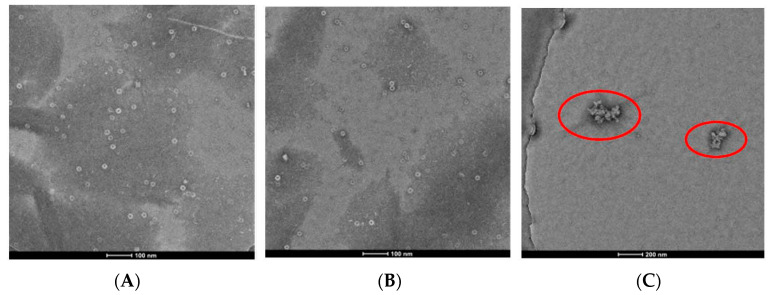
20S CP disk assembly inhibition of compound **3** (100 nm scale bar): (**A**) CP, (**B**) CP + 1/100 DMSO (100 nm scale bar), (**C**) CP + 10 μM compound **3** (200 nm scale bar).

Considering structural features and biological activity, compound **3** was chosen to study the mechanism of anti-TMV activity. The results showed that 20S CP and TMV RNA could assembled into TMV particles of about 300 nm in length, and dimethyl sulfoxide (DMSO) had no effect on the assembly (panels A and B of Figure 3). Compound **3** can cause a reduction in the length and number of TMV particles, indicating that it can inhibit the assembly of the viruses (panel C of Figure 3). The interaction experiment between compound **3** and 20S CP was also designed. The TMV protein can form the homogeneous-dispersed disc structure (Figure 4A), and a small amount of DMSO has no effect on the formation (Figure 4B). As seen in Figure 4C, compound **3** can lead to polymerization of TMV CP.

#### 3.4.2. Molecular Docking Study 

**Figure 5:**
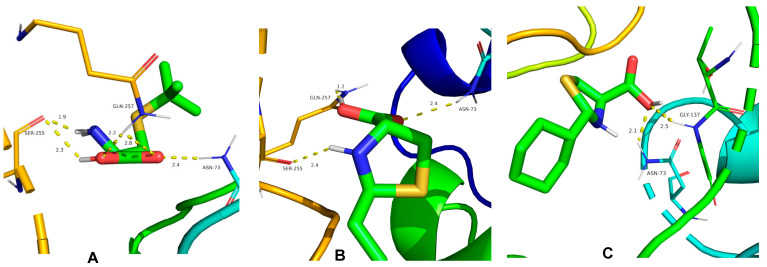
Molecule docking results of (**A**) compound **3**, (**B**) compound **23**, (**C**) compound **24** with TMV CP.

Molecular docking studies were performed to explore the binding sites of cysteine derivatives on TMV CP. Compounds **3**, **23**, and **24** were selected for molecular docking with TMV CP (PDB code 1EI7). The results showed that compound **3** has lain into the TMV CP activity pocket of SER 255, ASN 73, and GLN 257 (Figure 5A). Compound **3** forms five conventional hydrogen bonds with the active site of SER 255 (1.9 Å and 2.3 Å), GLN 257 (2.2 Å and 2.8 Å), and ASN 73 (2.4 Å) (Figure 5A). Compound **23** forms three conventional hydrogen bonds with the active site of SER 255 (2.4 Å), GLN 257 (1.2 Å), and ASN 73 (2.4 Å) (Figure 5B). As seen in Figure 5C, compound **24** forms two conventional hydrogen bonds with ASN 73 (2.1 Å) and GLY 137 (2.5 Å). The molecular docking results indicate that these compounds interact with CP through hydrogen bonding. The results of molecular docking also showed that compound **3** had more binding sites with TMV CP and shorter hydrogen bond distance. The stronger the interaction with TMV CP, the greater influence on the assembly of TMV, and the higher the inhibition rate. This result is consistent with the activity test.

### 3.5. Structure-Activity Relationship of the Fungicidal Activity

As the results showed in Table 3, all compounds exhibited broad-spectrum fungicidal activities in vitro. Kapachery et al. [30] reported that *N*-acetylcysteine (NAC, compound **7**) affected four kinds of bacteria (*Aeromonas hydrophila*, *Pseudomonas putida*, *Stenotrophomonas* sp., and *Serratia marcescens*) at the concentration of 1.5 mg/mL which were isolated from polluted reverse osmosis membrane. Perez-Giraldo et al. [31] confirmed that NAC could control bacterial biofilm formation on medical catheters. NAC has been widely used as a mucolytic agent in the treatment of chronic bronchitis [32]. However, the activity of cysteine and its derivatives on plant phytopathogenic fungi was not significant. The inhibitory effect of NAC on 14 plant pathogens was less than 50%. Changing the substituents of N, S, and O atoms in cysteine can slightly raise its anti-plant pathogen activity. Encouraged by the antifungal activities of compound **16**, compounds **1**–**24** were further evaluated in vivo fungicidal activity. The modification of cysteine had some effect on its fungicidal activity in vivo. It can be seen from the results of fungicidal activity that cysteine and its derivatives have broad-spectrum activity, but with a low to moderate degree of activity.

## 4. Materials and Methods

### 4.1. General Procedures

#### 4.1.1. Instruments

The melting points of the products were determined on an X-4 binocular microscope (Gongyi Yuhua Instrument Co., Gongyi, China) and are not corrected. NMR spectra were acquired with a 400 MHz (100 MHz for ^13^C) instrument (Bruker, Billerica, MA, USA) at room temperature. Chemical shifts were measured relative to residual solvent peaks of DMSO-*d*_6_ as internal standards (^1^H: δ = 2.5 and 3.3 ppm; ^13^C: δ = 39.9 ppm). The following abbreviations are used to designate chemical shift multiplicities: s = singlet, d = doublet, dd = doublet of doublets, t = triplet, m = multiplet, and brs = broad singlet. HRMS data were recorded with a QFT-ESI instrument (Varian, Palo Alto, CA, USA). All reagents were of analytical reagent (AR) grade or chemically pure (CR). Compounds **1**–**7** (AR) were purchased from Shanghai Bidepharm Co., Ltd. (Shanghai, China).

#### 4.1.2. Synthesis of Compounds **8**–**24**

l-Cysteine (3.63 g, 30 mmol) was dissolved in a mixed solvent of water (50 mL) and EtOH (6 mL). Then the solution of corresponding aldehydes (1.0 equiv.) in EtOH (15 mL) was added. The mixture was stirred at 25 °C for 6 h, filtered, washed with water, and dried to afford compounds **8**–**24** [7,26].

*(2RS,4R)-2-Phenyl-1,3-thiazolidine-4-carboxylic acid* (**8**). White solid, 93% yield, m.p. 155−157 °C (lit. [33] 158−159 °C); ^1^H-NMR (DMSO-*d*_6_): 7.25–7.52 (m, 5H, Ar-H), 5.67 (s, 0.5H, Ar-CH), 5.50 (s, 0.5H, Ar-CH), 4.24 (dd, *J* = 4.4 and 6.8 Hz, 0.5H, CH_2_CH), 3.90 (dd, *J* = 7.6 and 8.4 Hz, 0.5H, CH_2_CH), 3.38 (dd, *J* = 7.2 and 10.0 Hz, 0.5H, CH_2_), 3.30 (dd, *J* = 7.2 and 10.4 Hz, 0.5H, CH_2_), 3.14 (dd, *J* = 4.8 and 10.4 Hz, 0.5H, CH_2_), 3.08 (t, *J* = 8.8 Hz, 0.5H, CH_2_); ^13^C-NMR (DMSO-*d*_6_): 172.9, 172.2, 141.2, 138.9, 128.5, 128.3, 127.6, 127.2, 126.9, 71.7, 71.1, 65.4, 64.8, 38.4, 37.9; HRMS (ESI) *m/z* calc’d for C_10_H_11_NO_2_S [M + H]^+^: 209.0510, found 209.0503.

*(2RS,4R)-2-(4-Fluorophenyl)-1,3-thiazolidine-4-carboxylic acid* (**9**). White solid, 82% yield, m.p. 153–155 °C (lit. [34] 166 °C); ^1^H-NMR (DMSO-*d*_6_): 7.48–7.60 (m, 2H, Ar-H), 7.13–7.22 (m, 2H, Ar-H), 5.67 (s, 0.6H, Ar-CH), 5.51 (s, 0.4H, Ar-CH), 4.21 (dd, *J* = 4.8 and 6.8 Hz, 0.6H, CH_2_CH), 3.89 (t, *J* = 7.6 Hz, 0.4H, CH_2_CH), 3.36 (dd, *J* = 7.2 and 10.0 Hz, 0.5H, CH_2_), 3.30 (dd, *J* = 7.2 and 10.0 Hz, 0.7H, CH_2_), 3.13 (dd, *J* = 4.4 and 10.0 Hz, 0.6H, CH_2_), 3.09 (t, *J* = 9.2 Hz, 0.5H, CH_2_); ^13^C-NMR (DMSO-*d*_6_): 173.4, 172.6, 163.5, 163.1, 161.1, 160.7, 138.0, 135.8, 130.0, 129.9, 129.4, 115.8, 115.6, 115.5, 115.3, 71.4, 70.7, 66.0, 65.3, 38.8, 38.4; HRMS (ESI) *m/z* calc’d for C_10_H_10_FNO_2_S [M + H]^+^: 227.0416, found 227.0421.

*(2RS,4R)-2-(4-Bromophenyl)-1,3-thiazolidine-4-carboxylic acid* (**10**). White solid, 87% yield, m.p. 158–161 °C (lit. [34] 165–166 °C); ^1^H-NMR (DMSO-*d*_6_): 7.38–7.58 (m, 4H, Ar-H), 5.67 (s, 0.6H, Ar-CH), 5.49 (s, 0.4H, Ar-CH), 4.17 (dd, *J* = 4.8 and 6.8 Hz, 0.6H, CH_2_CH), 3.90 (dd, *J* = 7.2 and 8.8 Hz, 0.4H, CH_2_CH), 3.35 (dd, *J* = 6.8 and 10.0 Hz, 0.6H, CH_2_), 3.29 (dd, *J* = 7.2 and 10.4 Hz, 0.7H, CH_2_), 3.12 (t, *J* = 5.4 Hz, 0.6H, CH_2_), 3.08 (t, *J* = 7.2 Hz, 0.4H, CH_2_); ^13^C-NMR (DMSO-*d*_6_): 173.3, 172.5, 141.6, 139.1, 131.8, 131.6, 130.1, 129.6, 130.0, 71.3, 70.6, 66.0, 65.3, 38.8, 38.5; HRMS (ESI) *m/z* calc’d for C_10_H_10_BrNO_2_S [M + H]^+^: 288.9616, found 288.9614.

*(2RS,4R)-2-(4-Trifluoromethylphenyl)-1,3-thiazolidine-4-carboxylic acid* (**11**). White solid, 99% yield, m.p. 145–147 °C; ^1^H-NMR (DMSO-*d*_6_): 7.63–7.77 (m, 4H, Ar-H), 5.81 (s, 0.6H, Ar-CH), 5.61 (s, 0.4H, Ar-CH), 4.15 (t, *J* = 6.0 Hz, 0.6H, CH_2_CH), 3.95 (dd, *J* = 7.2 and 8.4 Hz, 0.4H, CH_2_CH), 3.37 (dd, *J* = 6.8 and 10.0 Hz, 0.4H, CH_2_), 3.31 (dd, *J* = 7.2 and 10.4 Hz, 0.6H, CH_2_), 3.08–3.12 (m, 1H, CH_2_); ^13^C-NMR (DMSO-*d*_6_): 173.2, 172.5, 147.3, 128.7, 128.0, 125.8, 125.7, 125.6, 71.1, 70.4, 66.1, 65.3, 38.6, 38.5; HRMS (ESI) *m/z* calc’d for C_11_H_10_F_3_NO_2_S [M +H]^+^: 277.0384, found 277.0383.

*(2RS,4R)-2-(4-Methoxyphenyl)-1,3-thiazolidine-4-carboxylic acid* (**12**). White solid, 93% yield, m.p. 153–156 °C (lit. [33] 163–164 °C); ^1^H-NMR (DMSO-*d*_6_): 7.44 (d, *J* = 8.8 Hz, 1H, Ar-H), 7.37 (d, *J* = 8.8 Hz, 1H, Ar-H), 6.92 (d, *J* = 8.8 Hz, 1H, Ar-H), 6.88 (d, *J* = 8.4 Hz, 1H, Ar-H), 5.60 (s, 0.5H, Ar-CH), 5.45 (s, 0.5H, Ar-CH), 4.25 (dd, *J* = 4.0 and 6.8 Hz, 0.5H, CH_2_CH), 3.87 (dd, *J* = 7.2 and 8.8 Hz, 0.5H, CH_2_CH), 3.75 (s, 1.5H, CH_3_), 3.74 (s, 1.5H, CH_3_), 3.36 (dd, *J* = 7.2 and 10.0 Hz, 0.5H, CH_2_), 3.28 (dd, *J* = 7.2 and 10.0 Hz, 0.5H, CH_2_), 3.15 (dd, *J* = 4.0 and 10.4 Hz, 0.5H, CH_2_), 3.07 (t, *J* = 8.8 Hz, 0.5H, CH_2_); ^13^C-NMR (DMSO-*d*_6_): 173.1, 172.3, 159.2, 158.7, 132.8, 130.7, 128.5, 128.3, 114.5, 113.8, 113.6, 71.5, 70.9, 65.4, 64.8, 55.1, 55.0, 38.5, 37.9; HRMS (ESI) *m/z* calc’d for C_11_H_13_NO_3_S [M + H]^+^: 239.0616, found 239.0612.

*(2RS,4R)-2-(4-Methylphenyl)-1,3-thiazolidine-4-carboxylic acid* (**13**). White solid, 99% yield, m.p. 146–148 °C (lit. [34] 163.2–163.7 °C); ^1^H-NMR (DMSO-*d*_6_): 7.39 (d, *J* = 8.0 Hz, 1H, Ar-H), 7.32 (d, *J* = 8.0 Hz, 1H, Ar-H), 7.17 (d, *J* = 7.6 Hz, 1H, Ar-H), 7.13 (d, *J* = 7.6 Hz, 1H, Ar-H), 5.61 (s, 0.5H, Ar-CH), 5.46 (s, 0.5H, Ar-CH), 4.24 (dd, *J* = 4.4 and 6.8 Hz, 0.5H, CH_2_CH), 3.87 (dd, *J* = 7.6 and 8.8 Hz, 0.5H, CH_2_CH), 3.36 (dd, *J* = 6.8 and 10.0 Hz, 0.5H, CH_2_), 3.28 (dd, *J* = 6.8 and 10.0 Hz, 0.5H, CH_2_), 3.14 (dd, *J* = 4.0 and 10.0 Hz, 0.5H, CH_2_), 3.07 (t, *J* = 8.8 Hz, 0.5H, CH_2_), 2.30 (s, 1.5H, CH_3_), 2.28 (s, 1.5H, CH_3_); ^13^C-NMR (DMSO-*d*_6_): 173.5, 172.8, 138.5, 138.1, 137.3, 136.3, 130.2, 130.1, 129.4, 129.2, 127.6, 127.4, 72.2, 71.6, 65.9, 65.3, 38.9, 38.4, 21.2, 21.1; HRMS (ESI) *m/z* calc’d for C_11_H_13_NO_2_S [M + H]^+^: 223.0667, found 223.0673.

*(2RS,4R)-2-(4-Hydroxyphenyl)-1,3-thiazolidine-4-carboxylic acid* (**14**). White solid, 81% yield, m.p. 161–164 °C (lit. [35] 167–1169 °C); ^1^H-NMR (DMSO-*d*_6_): 9.51 (br s, 1H, OH), 7.33 (d, *J* = 8.8 Hz, 1H, Ar-H), 7.26 (d, *J* = 8.0 Hz, 1H, Ar-H), 6.75 (d, *J* = 8.0 Hz, 1H, Ar-H), 6.72 (d, *J* = 8.8 Hz, 1H, Ar-H), 5.55 (s, 0.5H, Ar-CH), 5.41 (s, 0.5H, Ar-CH), 4.26 (dd, *J* = 3.6 and 6.8 Hz, 0.5H, CH_2_CH), 3.86 (t, *J* = 7.6 Hz, 0.5H, CH_2_CH), 3.36 (dd, *J* = 6.8 and 10.0 Hz, 0.5H, CH_2_), 3.28 (dd, *J* = 6.8 and 10.0 Hz, 0.5H, CH_2_), 3.16 (dd, *J* = 3.6 and 10.4 Hz, 0.5H, CH_2_), 3.06 (t, *J* = 9.2 Hz, 0.5H, CH_2_); ^13^C-NMR (DMSO-*d*_6_): 173.1, 172.3, 157.4, 156.9, 130.7, 128.8, 128.5, 128.3, 115.1, 114.9, 71.8, 71.2, 65.2, 64.7, 38.5, 37.8; HRMS (ESI) *m/z* calc’d for C_10_H_11_NO_3_S [M + H]^+^: 225.0460, found 225.0463.

*(2RS,4R)-2-(3-Hydroxy-4-methoxyphenyl)-1,3-thiazolidine-4-carboxylic acid* (**15**). White solid, 93% yield, m.p. 170–173 °C; ^1^H-NMR (DMSO-*d*_6_): 9.03 (br s, 1H, OH), 7.12 (s, 0.5H, Ar-H), 7.11 (s, 0.5H, Ar-H), 6.83–6.90 (m, 1H, Ar-H), 6.69–6.74 (m, 1H, Ar-H), 5.53 (s, 0.5H, Ar-CH), 5.40 (s, 0.5H, Ar-CH), 4.29 (dd, *J* = 3.6 and 6.8 Hz, 0.5H, CH_2_CH), 3.83 (dd, *J* = 7.2 and 8.8 Hz, 0.5H, CH_2_CH), 3.77 (s, 1.5H), 3.76 (s, 0.5H, CH_2_CH), 3.33 (dd, *J* = 7.2 and 10.0 Hz, 0.5H, CH_2_), 3.28 (dd, *J* = 7.2 and 10.0 Hz, 0.5H, CH_2_), 3.16 (dd, *J* = 3.6 and 10.4 Hz, 0.5H, CH_2_), 3.06 (t, *J* = 9.2 Hz, 0.5H, CH_2_); ^13^C-NMR (DMSO-*d*_6_): 173.7, 172.8, 148.0, 147.8, 147.1, 146.6, 131.7, 129.9, 120.4, 120.1, 115.6, 115.4, 111.9, 111.7, 72.6, 72.0, 66.0, 65.3, 56.1, 56.0, 38.9, 38.3; HRMS (ESI) *m/z* calc’d for C_11_H_13_NO_4_S [M + H]^+^: 255.0565, found 255.0573.

*(2RS,4R)-2-(4-Nitrophenyl)-1,3-thiazolidine-4-carboxylic acid* (**16**). White solid, 99% yield, m.p. 94–97 °C (lit. [34] 95–97 °C); ^1^H-NMR (DMSO-*d*_6_): 8.17–8.23 (m, 2H, Ar-H), 7.80 (d, *J* = 8.8 Hz, 0.8H, Ar-H), 7.67 (d, *J* = 8.8 Hz, 1H, Ar-H), 5.87 (s, 0.6H, Ar-CH), 5.66 (s, 0.4H, Ar-CH), 4.12 (t, *J* = 6.4 Hz, 0.7H, CH_2_CH), 3.97 (dd, *J* = 6.8 and 9.2 Hz, 0.5H, CH_2_CH), 3.37 (dd, *J* = 6.8 and 10.0 Hz, 0.7H, CH_2_), 3.32 (dd, *J* = 6.8 and 10.0 Hz, 0.8H, CH_2_), 3.06–3.13 (m, 1H, CH_2_); ^13^C-NMR (DMSO-*d*_6_): 173.1, 172.5, 150.6, 147.7, 147.1, 129.1, 128.3, 124.0 123.9, 70.6, 69.9, 66.2, 65.4, 38.6, 38.5; HRMS (ESI) *m/z* calc’d for C_10_H_10_N_2_O_4_S [M + H]^+^: 254.0361, found 254.0367.

*(2RS,4R)-2-(3-Nitrophenyl)-1,3-thiazolidine-4-carboxylic acid* (**17**). White solid, 99% yield, m.p. 90–93 °C; ^1^H-NMR (DMSO-*d*_6_): 8.46 (s, 0.4H, Ar-H), 8.29 (s, 0.6H, Ar-H), 8.17 (dd, *J* = 1.6 and 8.0 Hz, 0.4H, Ar-H), 8.12 (dd, *J* = 1.6 and 8.4 Hz, 0.6H, Ar-H), 7.96 (d, *J* = 8.0 Hz, 0.4H, Ar-H), 7.87 (d, *J* = 7.6 Hz, 0.6H, Ar-H), 7.60–7.67 (m, 1H, Ar-H), 5.86 (s, 0.6H, Ar-CH), 5.67 (s, 0.4H, Ar-CH), 4.14 (t, *J* = 6.0 Hz, 0.6H), 3.95 (dd, *J* = 7.2 and 8.4 Hz, 0.5H, CH_2_CH), 3.31–3.38 (m, 1H, CH_2_), 3.09–3.15 (m, 1H, CH_2_); ^13^C-NMR (DMSO-*d*_6_): 173.1, 172.4, 148.2, 148.1, 145.1, 142.5, 134.8, 134.3, 130.4, 130.3, 70.6, 69.9, 66.3, 65.2, 38.6, 38.5; HRMS (ESI) *m/z* calc’d for C_10_H_10_N_2_O_4_S [M + H]^+^: 254.0361, found 254.0365.

*(2RS,4R)-2-(Thiophen-2-yl)thiazolidine-4-carboxylic acid* (**18**). White solid, 99% yield, m.p. 144–147 °C (lit. [36] 146–147 °C); ^1^H-NMR (DMSO-*d*_6_): 7.51 (dd, *J* = 0.8 and 4.8 Hz, 0.3H, Ar-H), 7.42 (dd, *J* = 0.8 and 5.2 Hz, 0.6H, Ar-H), 7.20 (d, *J* = 3.2 Hz, 0.4H, Ar-H), 7.06 (d, *J* = 3.6 Hz, 0.6H, Ar-H), 6.99 (dd, *J* = 3.2 and 4.8 Hz, 0.3H, Ar-H), 6.95 (dd, *J* = 3.6 and 4.2 Hz, 0.6H, Ar-H), 5.94 (s, 0.7H, Ar-CH), 5.75 (s, 0.4H, Ar-CH), 4.07 (t, *J* = 6.4 Hz, 0.7H, CH_2_CH), 3.91 (dd, *J* = 7.2 and 8.8 Hz, 0.4H, CH_2_CH), 3.31–3.39 (m, 1H, CH_2_), 3.03–3.11 (m, 1H, CH_2_); ^13^C-NMR (DMSO-*d*_6_): 172.6, 172.2, 147.1, 142.8, 126.8, 126.7, 126.2, 125.4, 125.2, 66.6, 66.1, 65.4, 64.5, 38.5, 38.0; HRMS (ESI) *m/z* calc’d for C_8_H_9_NO_2_S_2_ [M + H]^+^: 215.0075, found 215.0079.

*(2RS,4R)-2-(Furan-2-yl)thiazolidine-4-carboxylic acid* (**19**). White solid, 99% yield, m.p. 137–140 °C (lit. [33] 137–138 °C); ^1^H-NMR (DMSO-*d*_6_): 7.65–7.66 (m, 0.3H, Ar-H), 7.58–7.59 (m, 0.6H, Ar-H), 6.50 (d, *J* = 3.2 Hz, 0.3H, Ar-H), 6.43–6.44 (m, 0.3H, Ar-H), 6.37–6.38 (m, 0.6H, Ar-H), 6.34–6.35 (m, 0.6H, Ar-H), 5.74 (s, 0.7H, Ar-CH), 5.61 (s, 0.4H, Ar-CH), 4.11 (t, *J* = 6.4 Hz, 0.7H, CH_2_CH), 3.87 (dd, *J* = 6.8 and 8.8 Hz, 0.4H, CH_2_CH), 3.27–3.37 (m, 1H, CH_2_), 2.97–3.01 (m, 1H, CH_2_); ^13^C-NMR (DMSO-*d*_6_): 173.0, 172.7, 154.9, 151.8, 143.4, 142.9, 111.1, 110.8, 108.0, 106.8, 65.9, 65.3, 64.7, 64.4, 38.6, 38.3; HRMS (ESI) *m/z* calc’d for C_8_H_9_NO_3_S [M + H]^+^: 119.0303, found 119.0305.

*(2RS,4R)-2-(Pyridin-4-yl)thiazolidine-4-carboxylic acid* (**20**). White solid, 93% yield, m.p. 163–165 °C (lit. [34] 175–176 °C); ^1^H-NMR (DMSO-*d*_6_): 8.55 (d, *J* = 6.0 Hz, 0.4H, Ar-H), 8.50 (d, *J* = 6.1 Hz, 1.6H, Ar-H), 7.52 (d, *J* = 6.0 Hz, 0.4H, Ar-H), 7.40 (d, *J* = 6.0 Hz, 1.6H, Ar-H), 5.75 (s, 0.8H, Ar-CH), 5.54 (s, 0.2H, Ar-CH), 4.10 (t, *J* = 6.3 Hz, 0.8H, CH_2_CH), 3.97 (dd, *J* = 6.9 and 8.8 Hz, 0.2H, CH_2_CH), 3.36 (dd, *J* = 6.9 and 10.0 Hz, 0.2H, CH_2_), 3.30 (dd, *J* = 6.8 and 10.2 Hz, 0.8H, CH_2_), 3.04–3.10 (m, 1H, CH_2_); ^13^C-NMR (DMSO-*d*_6_): 173.0, 172.5, 151.9, 151.1, 150.1, 149.8, 122.3, 122.1, 70.2, 69.5, 66.2, 65.3, 38.6, 38.4; HRMS (ESI) *m/z* calc’d for C_9_H_10_N_2_O_2_S [M + H]^+^: 210.0463, found 210.0460.

*(2RS,4R)-2-Benzylthiazolidine-4-carboxylic acid* (**21**). White solid, 93% yield, m.p. 152–155 °C (lit. [35] 165–166 °C); ^1^H-NMR (DMSO-*d*_6_): 7.18–7.30 (m, 5H, Ar-H), 4.81 (t, *J* = 7.0 Hz, 0.7H, SCH), 4.65 (t, *J* = 6.8 Hz, 0.3H, SCH), 4.15 (dd, *J* = 5.6 and 6.7 Hz, 0.7H, CH_2_CH), 3.72 (dd, *J* = 6.9 and 9.2 Hz, 0.3H, CH_2_CH), 3.07–3.27 (m, 2H, CH_2_CH and Ar-CH_2_), 3.00 (dd, *J* = 7.3 and 13.7 Hz, 0.3H, CH_2_CH), 2.92 (dd, *J* = 5.4 and 10.2 Hz, 0.7H, CH_2_CH), 2.75–2.85 (m, 1H, Ar-CH_2_); ^13^C-NMR (DMSO-*d*_6_): 173.3, 172.7, 139.5, 139.3, 129.6, 129.4, 128.7, 128.6, 126.9, 126.7, 72.4, 71.8, 65.7, 64.6, 43.3, 41.1, 37.8, 37.6; HRMS (ESI) *m/z* calc’d for C_11_H_13_NO_2_S [M + H]^+^: 223.0667, found 223.0662.

*(2RS,4R)-2-Propylthiazolidine-4-carboxylic acid* (**22**). White solid, 93% yield, m.p. 190–192 °C; ^1^H-NMR (DMSO-*d*_6_): 4.56 (t, *J* = 6.6 Hz, 0.5H, SCH), 4.42 (t, *J* = 7.2 Hz, 0.5H, SCH), 4.07 (dd, *J* = 6.7 Hz and 5.4 Hz, 0.5H, SCH_2_CH), 3.71 (dd, *J* = 7.0 and 9.0 Hz, 0.5H, SCH_2_CH), 3.20 (dd, *J* = 7.0 and 9.9 Hz, 0.5H, SCH_2_), 3.09 (dd, *J* = 7.0 and 10.2 Hz, 0.5H, SCH_2_), 2.94 (dd, *J* = 5.1 and 10.2 Hz, 0.5H, SCH_2_), 2.76 (t, *J* = 9.4 Hz, 0.5H, SCH_2_), 1.85–1.93 (m, 0.5H, CH_2_CH_2_CH), 1.65–1.77 (m, 1H, CH_2_CH_2_CH), 1.49–1.56 (m, 0.5H, CH_2_CH_2_CH), 1.35–1.43 (m, 2H, CH_2_CH_2_), 0.86–0.91 (m, 3H, CH_3_); ^13^C-NMR (DMSO-*d*_6_): 173.3, 172.8, 71.2, 70.4, 65.6, 64.6, 39.3, 39.2, 37.4, 37.0, 21.4, 21.1, 14.3, 14.2; HRMS (ESI) *m/z* calc’d for C_7_H_13_NO_2_S [M + H]^+^: 175.0667, found 175.0670.

*(2RS,4R)-2-Heptylthiazolidine-4-carboxylic acid* (**23**). White solid, 93% yield, m.p. 136–139 °C (lit. [37] 159–160 °C); ^1^H-NMR (DMSO-*d*_6_): 4.54 (t, *J* = 6.6 Hz, 0.5H, SCH), 4.40 (t, *J* = 6.7 Hz, 0.5H, SCH), 4.06 (t, *J* = 6.1 Hz, 0.5H, SCH_2_CH), 3.70 (t, *J* = 8.5 Hz, 0.5H, SCH_2_CH), 3.19 (t, *J* = 9.6 Hz, 0.5H, SCH_2_), 3.08 (t, *J* = 9.8 Hz, 0.5H, SCH_2_), 2.93 (dd, *J* = 4.9 and 9.8 Hz, 0.5H, SCH_2_), 2.75 (t, *J* = 9.6 Hz, 0.5H, SCH_2_), 1.85–1.92 (m, 0.5H, CH_2_CH_2_CH), 1.69–1.76 (m, 1H, CH_2_CH_2_CH), 1.50–1.67 (m, 0.5H, CH_2_CH_2_CH), 1.24–1.48 (m, 10H, CH_2_CH_2_), 0.84–0.86 (m, 3H, CH_3_); ^13^C-NMR (DMSO-*d*_6_): 173.3, 172.8, 71.5, 70.8, 65.7, 64.6, 37.4, 37.1, 37.0, 35.3, 31.7, 29.3, 29.2, 29.1, 29.0, 28.1, 27.9, 22.5, 14.4; HRMS (ESI) *m/z* calc’d for C_11_H_21_NO_2_S [M + H]^+^: 231.1293, found 231.1294.

*(2RS,4R)-2-Cyclohexylthiazolidine-4-carboxylic acid* (**24**). White solid, 82% yield, m.p.184–187 °C; ^1^H-NMR (DMSO-*d*_6_): 4.01–4.02 (m, 0.4H, SCH), 3.65–3.69 (m, 0.6H, SCH), 3.13–3.16 (m, 0.6H, SCH_2_CH), 2.98–3.01 (m, 0.4H, SCH_2_CH), 2.87–2.89 (m, 0.4H, SCH_2_CH), 2.65–2.68 (m, 0.6H, SCH_2_CH), 1.95–1.97 (m, 1H, CH_2_CHCH_2_), 1.40–1.66 (m, 4H, CH_2_CH_2_CH), 0.98–1.15 (m, 6H, CH_2_CH_2_CH_2_); ^13^C-NMR (DMSO-*d*_6_): 173.4, 172.9, 77.4, 76.6, 65.7, 64.8, 44.1, 43.1, 36.7, 31.7, 31.6, 30.1, 26.4, 26.0, 25.9, 25.8; HRMS (ESI) *m/z* calc’d for C_10_H_17_NO_2_S [M + H]^+^: 215.0980, found 215.0978.

### 4.2. Biological Assays

Each test was repeated three times at 25 ± 1 °C. Active effect expressed in percentage scale of 0–100 (0: no activity; 100: total inhibited). Specific test methods for the anti-TMV and fungicidal activities were carried out by the literature method [5,27], Detailed bioassay procedures for the anti-TMV and fungicidal activities were described in the literature and can be seen in the Supplementary Materials.

## 5. Conclusions

Based on the structure of natural product cysteine, a series of cysteine and its derivatives were designed, synthesized, and evaluated for their antiviral and antifungal activities in vitro and in vivo. By studying the influence of O, N, and S atom substituents of cysteine, it was found that some compounds had excellent anti-TMV activity. The preliminary mode of action studies exhibited that compound **3** can hold back virus assembly by aggregating the 20S protein disk. We further study the binding sites of the interaction between cysteines and TMV CP by molecular docking. Further fungicidal activity tests against 14 kinds of phytopathogenic fungi revealed that these cysteine derivatives displayed broad-spectrum fungicidal activities. In this work, cysteine and its derivatives are found to be potential inhibitors against plant viruses and plant pathogens.

## Figures and Tables

**Table 1:** In vitro antiviral activity of compounds **1**–**24** against TMV.

Compd.	Concn (μg/mL)	Inhibition Rate (%) ^a^	Compd.	Concn (μg/mL)	Inhibition Rate (%) ^a^
**1-D**	500	39 ± 3	**13**	500	43 ± 1
	100	13 ± 2		100	17 ± 1
**1-L**	500	41 ± 2	**14**	500	18 ± 2
	100	11 ± 1		100	0
**2-D**	500	38 ± 2	**15**	500	34 ± 1
	100	15 ± 2		100	12 ± 1
**2-L**	500	40 ± 1	**16**	500	40 ± 2
	100	16 ± 2		100	16 ± 2
**3**	500	48 ± 1	**17**	500	39 ± 1
	100	13 ± 2		100	14 ± 2
**4**	500	45 ± 2	**18**	500	24 ± 2
	100	12 ± 1		100	0
**5**	500	42 ± 2	**19**	500	32 ± 2
	100	10 ± 1		100	0
**6**	500	40 ± 2	**20**	500	43 ± 2
	100	20 ± 1		100	18 ± 2
**7**	500	44 ± 2	**21**	500	35 ± 2
	100	22 ± 1		100	11 ± 1
**8**	500	33 ± 2	**22**	500	33 ± 1
	100	9 ± 2		100	10 ± 1
**9**	500	35 ± 1	**23**	500	46 ± 1
	100	12 ± 2		100	17 ± 2
**10**	500	39 ± 2	**24**	500	45 ± 1
	100	13 ± 1		100	19 ± 1
**11**	500	43 ± 1	Ribavirin	500	35 ± 1
	100	19 ± 1		100	7 ± 1
**12**	500	38 ± 2	Ningnanmycin	500	61 ± 2
	100	16 ± 1		100	23 ± 2

^a^ Average of three replicates. All results are expressed as mean ± SD.

**Table 2:** In Vivo antiviral activity of compounds **1**–**24** against TMV.

Compd.	Concn (μg/mL)	Inactivation Effect (%) ^a^	Curative Effect (%) ^a^	Protection Effect (%) ^a^
**1-D**	500	44 ± 2	38 ± 2	37 ± 1
	100	18 ± 3	15 ± 2	14 ± 1
**1-L**	500	43 ± 1	42 ± 2	40 ± 1
	100	20 ± 2	16 ± 1	13 ± 2
**2-D**	500	37 ± 2	41 ± 3	36 ± 1
	100	12 ± 1	13 ± 1	15 ± 2
**2-L**	500	39 ± 1	40 ± 2	38 ± 2
	100	11 ± 2	14 ± 1	12 ± 1
**3**	500	51 ± 2	47 ± 2	49 ± 2
	100	26 ± 1	23± 1	25 ± 1
**4**	500	45 ± 1	43 ± 1	46 ± 1
	100	18 ± 2	20 ± 2	22 ± 2
**5**	500	40 ± 1	39 ± 2	37 ± 2
	100	13 ± 1	16 ± 1	16 ± 1
**6**	500	38 ± 2	40 ± 2	35 ± 2
	100	16 ± 2	16 ± 1	16 ± 1
**7**	500	44 ± 1	42 ± 2	41 ± 2
	100	20 ± 2	16 ± 1	18 ± 1
**8**	500	37 ± 2	33 ± 2	35 ± 2
	100	11 ± 1	10 ± 1	11 ± 1
**9**	500	36 ± 2	35 ± 1	34 ± 1
	100	10 ± 1	9 ± 1	9 ± 1
**10**	500	41 ± 2	40 ± 2	41 ± 2
	100	10 ± 1	10 ± 1	13 ± 1
**11**	500	40 ± 2	39 ± 2	40 ± 2
	100	15 ± 1	17 ± 1	12 ± 1
**12**	500	43 ± 2	36 ± 2	41 ± 2
	100	14 ± 1	13 ± 1	15 ± 1
**13**	500	49 ± 2	46 ± 2	44 ± 2
	100	18 ± 2	15 ± 2	13 ± 2
**14**	500	21 ± 2	17 ± 2	15 ± 2
	100	0	0	0
**15**	500	28 ± 2	33 ± 2	25 ± 2
	100	9 ± 1	11 ± 1	7 ± 1
**16**	500	42 ± 1	37 ± 1	35 ± 1
	100	0	0	0
**17**	500	40 ± 1	36 ± 1	35 ± 1
	100	0	0	0
**18**	500	24 ± 2	23 ± 2	20 ± 2
	100	0	0	0
**19**	500	34 ± 2	30 ± 2	36 ± 2
	100	0	0	0
**20**	500	47 ± 2	45 ± 2	43 ± 2
	100	21 ± 2	19 ± 2	14 ± 2
**21**	500	37 ± 2	40 ± 2	34 ± 2
	100	11 ± 2	15 ± 2	11 ± 2
**22**	500	33 ± 2	37 ± 2	32 ± 2
	100	10 ± 2	13 ± 2	9 ± 2
**23**	500	48 ± 1	49 ± 1	47 ± 1
	100	19 ± 2	15 ± 2	17 ± 2
**24**	500	47 ± 1	42 ± 1	43 ± 1
	100	15 ± 1	17 ± 1	15 ± 1
Ribavirin	500	40 ± 1	40 ± 1	38 ± 1
	100	13 ± 1	15 ± 1	10 ± 1
Ningnanmycin	500	56 ± 2	53 ± 2	59 ± 1
	100	28 ± 1	24 ± 1	30 ± 1

^a^ Average of three replicates. All results are expressed as mean ± SD.

**Table 3:** In vitro fungicidal activities of compounds **1**–**24** against 14 kinds of fungi.

Compd.	Fungicidal Activity (%) ^a^ at 50 μg/mL													
	*F.C* ^b^	*C.H* ^b^	*P.P* ^b^	*R.C* ^b^	*B.M* ^b^	*W.A* ^b^	*F.M* ^b^	*A.S* ^b^	*F.G* ^b^	*P.I* ^b^	*P.C* ^b^	*S.S* ^b^	*B.C* ^b^	*R.S* ^b^
**1-D**	18 ± 2	7 ± 1	30 ± 2	9 ± 1	10 ± 2	11 ± 3	9 ± 1	37 ± 3	16 ± 2	8 ± 2	27 ± 3	20 ± 1	16 ± 2	11 ± 1
**1-L**	16 ± 1	5 ± 1	35 ± 1	11 ± 1	7 ± 2	16 ± 1	6 ± 1	35 ± 1	10 ± 2	10 ± 1	22 ± 1	25 ± 1	18 ± 1	12 ± 1
**2-D**	13 ± 1	19 ± 2	25 ± 2	12 ± 1	26 ± 2	30 ± 2	16 ± 1	42 ± 1	24 ± 1	13 ± 1	16 ± 1	31 ± 3	24 ± 1	17 ± 2
**2-L**	18 ± 1	13 ± 2	29 ± 2	15 ± 1	21 ± 2	33 ± 2	19 ± 1	49 ± 2	26 ± 1	13 ± 1	17 ± 1	36 ± 2	21 ± 1	14 ± 1
**3**	11 ± 1	13 ± 1	22 ± 1	26 ± 2	10 ± 1	11 ± 1	13 ± 1	39 ± 1	13 ± 1	15 ± 1	21 ± 1	23 ± 1	17 ± 1	16 ± 1
**4**	9 ± 1	7 ± 1	31 ± 1	16 ± 1	13 ± 2	22 ± 1	16 ± 1	36 ± 1	13 ± 2	15 ± 2	12 ± 1	19 ± 1	10 ± 1	17 ± 2
**5**	23 ± 1	16 ± 2	35 ± 1	10 ± 1	23 ± 2	16 ± 1	24 ± 1	42 ± 1	10 ± 1	13 ± 1	17 ± 1	29 ± 1	14 ± 1	13 ± 2
**6**	12 ± 1	18 ± 1	41 ± 1	36 ± 1	26 ± 2	19 ± 2	15 ± 1	43 ± 1	13 ± 1	11 ± 1	17 ± 1	21 ± 1	8 ± 1	13 ± 1
**7**	37 ± 1	15 ± 1	32 ± 1	42 ± 1	25 ± 1	19 ± 1	23 ± 1	46 ± 1	17 ± 1	13 ± 1	34 ± 1	19 ± 1	22 ± 1	17 ± 1
**8**	10 ± 1	14 ± 1	14 ± 1	9 ± 1	7 ± 1	16 ± 1	6 ± 2	42 ± 1	42 ± 1	16 ± 1	12 ± 1	19 ± 2	14 ± 1	6 ± 1
**9**	16 ± 1	14 ± 1	17 ± 1	5 ± 1	17 ± 1	12 ± 1	18 ± 1	39 ± 1	32 ± 2	10 ± 2	6 ± 1	25 ± 1	13 ± 1	8 ± 1
**10**	29 ± 1	52 ± 2	62 ± 1	28 ± 1	14 ± 2	32 ± 1	18 ± 2	39 ± 1	32 ± 1	10 ± 1	12 ± 1	13 ± 1	9 ± 1	16 ± 1
**11**	19 ± 2	14 ± 1	24 ± 1	12 ± 1	7 ± 1	16 ± 1	18 ± 2	39 ± 2	13±	10 ± 1	10 ± 1	31 ± 1	4 ± 1	23 ± 1
**12**	29 ± 1	19 ± 1	10 ± 1	11 ± 2	14 ± 2	24 ± 2	24 ± 1	23 ± 1	10 ± 1	13 ± 2	14 ± 1	19 ± 2	13 ± 1	12 ± 1
**13**	26 ± 1	10 ± 1	38 ± 2	9 ± 1	17 ± 1	16 ± 1	12 ± 1	39 ± 1	10 ± 1	3 ± 1	12 ± 1	25 ± 1	11 ± 1	6 ± 1
**14**	13 ± 1	0	17 ± 1	0	10 ± 1	12 ± 1	6 ± 1	42 ± 1	16 ± 1	7 ± 1	8 ± 1	25 ± 1	13 ± 1	8 ± 1
**15**	19 ± 2	10 ± 2	20 ± 2	18 ± 1	7 ± 1	16 ± 2	12 ± 2	27 ± 1	10 ± 1	13 ± 1	4 ± 1	19 ± 1	9 ± 1	12 ± 1
**16**	32 ± 1	71 ± 1	83 ± 1	54 ± 1	45 ± 1	16 ± 2	18 ± 1	58 ± 1	32 ± 1	10 ± 1	31 ± 2	50 ± 1	48 ± 1	29 ± 1
**17**	16 ± 1	10 ± 1	38 ± 1	18 ± 1	10 ± 1	20 ± 1	12 ± 1	31 ± 1	10 ± 1	13 ± 2	16 ± 1	44 ± 2	18 ± 1	8 ± 1
**18**	16 ± 1	48 ± 1	45 ± 2	23 ± 2	10 ± 1	20 ± 1	18 ± 1	27 ± 1	19 ± 1	10 ± 1	20 ± 1	31 ± 1	7 ± 1	12 ± 1
**19**	48 ± 2	33 ± 1	45 ± 1	19 ± 1	10 ± 1	36 ± 1	35 ± 1	35 ± 2	13 ± 2	7 ± 1	29 ± 1	13 ± 1	11 ± 1	6 ± 1
**20**	26 ± 1	10 ± 1	21 ± 1	12 ± 1	3 ± 2	16 ± 1	12 ± 1	39 ± 2	16 ± 1	10 ± 1	6 ± 1	19 ± 1	7 ± 1	6 ± 1
**21**	19 ± 2	0	17 ± 1	28 ± 1	14 ± 2	20 ± 1	24 ± 1	46 ± 1	32 ± 1	10 ± 2	22 ± 1	25 ± 1	4 ± 1	12 ± 1
**22**	16 ± 1	10 ± 1	31 ± 1	14 ± 1	7 ± 1	12 ± 1	12 ± 2	39 ± 1	32 ± 1	16 ± 1	16 ± 1	19 ± 1	21 ± 1	12 ± 1
**23**	23 ± 1	19 ± 1	21 ± 1	9 ± 1	10 ± 1	16 ± 1	29 ± 1	35 ± 1	16 ± 1	13 ± 1	6 ± 1	31 ± 1	21 ± 1	12 ± 1
**24**	19 ± 1	19 ± 1	35 ± 1	12 ± 1	7 ± 1	16 ± 1	18 ± 1	50 ± 1	42 ± 1	3 ± 1	6 ± 1	31 ± 1	27 ± 2	14 ± 1
Chlorothalonil ^c^	100	69 ± 1	89 ± 1	100	91 ± 1	95 ± 1	100	56 ± 1	100	100	55 ± 2	100	100	100
Carbendazim ^c^	100	53 ± 1	100	100	100	100	71 ± 1	56 ± 2	88 ± 2	83 ± 1	90 ± 2	100	96 ± 1	100

^a^ Average of three replicates. All results are expressed as mean ± SD. ^b^ *F.C, Fusarium oxysporium* f. sp. *Cucumeris, C.H, Cercospora arachidicola Hori, P.P, Physalospora piricola, R.C, Rhizoctonia cerealis, B.M, Bipolaris maydis, W.A, Watermelon anthracnose, F.M, Fusarium moniliforme, A.S, Alternaria solani, F.G, Fusarium graminearum, P.I, Phytophthora infestans, P.C, Phytophthora capsica, S.S, Sclerotinia sclerotiorum, B.C, Botrytis cinereal, R.S, Rhizoctonia solani*. ^c^ The commercial agricultural fungicides were used for comparison of antifungal activity.

**Table 4:** In vivo fungicidal activities of compounds **1**–**24** against six kinds of fungi.

Compd.	Inhibition Rate ^a^ (%) at 200 μg/mL					
	*B.G* ^b^	*S.S* ^b^	*B.C* ^b^	*R.S* ^b^	*C.C* ^b^	*P.C* ^b^
**1-D**	0	12 ± 2	5 ± 1	9 ± 2	10 ± 1	5 ± 1
**1-L**	0	11 ± 1	7 ± 2	7 ± 1	11 ± 2	5 ± 1
**2-D**	0	8 ± 2	12 ± 2	8 ± 2	13 ± 2	12 ± 1
**2-L**	0	10 ± 2	11 ± 1	7 ± 2	17 ± 2	13 ± 1
**3**	0	7 ± 2	13 ± 1	8 ± 2	13 ± 1	13 ± 1
**4**	0	10 ± 1	11 ± 2	0	13 ± 2	0
**5**	0	19 ± 3	4 ± 1	11 ± 1	5 ± 1	12 ± 2
**6**	0	16 ± 1	15 ± 2	8 ± 2	11 ± 1	7 ± 1
**7**	0	10 ± 2	12 ± 1	16 ± 1	13 ± 2	5 ± 1
**8**	0	4 ± 1	9 ± 1	0	6 ± 2	5 ± 1
**9**	0	11 ± 1	5 ± 1	0	0	0
**10**	0	11 ± 3	12 ± 2	14 ± 1	10 ± 1	0
**11**	0	16 ± 1	1 ± 1	11 ± 3	17 ± 3	0
**12**	0	11 ± 3	7 ± 3	20 ± 1	13 ± 2	5 ± 1
**13**	0	17 ± 1	6 ± 1	0	13 ± 2	5 ± 1
**14**	0	11 ± 2	7 ± 1	0	11 ± 1	0
**15**	0	11 ± 2	5 ± 1	11 ± 2	4 ± 1	0
**16**	0	19 ± 1	26 ± 1	16 ± 2	13 ± 1	10 ± 1
**17**	0	17 ± 2	15 ± 2	0	0	5 ± 2
**18**	0	4 ± 1	3 ± 1	20 ± 1	26 ± 1	5 ± 1
**19**	20 ± 2	4 ± 1	11 ± 2	0	20 ± 2	10 ± 2
**20**	0	17 ± 2	11 ± 2	0	20 ± 1	0
**21**	0	7 ± 1	12 ± 2	0	11 ± 2	5 ± 1
**22**	0	16 ± 2	15 ± 2	5 ± 1	8 ± 1	0
**23**	0	10 ± 2	9 ± 1	0	12 ± 2	5 ± 1
**24**	0	11 ± 1	15 ± 2	0	22 ± 1	0
azoxystrobin ^c^	81 ± 2	100	100	75 ± 2	80 ± 1	85 ± 2

^a^ Average of three replicates. All results are expressed as mean ± SD. ^b^ *B.G, Blumeria graminis* f.sp. *tritici, S.S, Sclerotinia sclerotiorum, B.C, Botrytis cinereal, R.S, Rhizoctonia solani, C.C, Corynespora cassiicola, P.C, Phytophthora capsica*. ^c^ The commercial agricultural fungicides were used for comparison of antifungal activity.

## Data Availability

Not applicable.

## Supplementary Materials

The following are available online, Supplementary data (Detailed bio-assay procedures for the anti-TMV and fungicidal activities; mode of action studies; copies of ^1^H & ^13^C-NMR spectra) can be found in the online version.

## Sample Availability

Samples of the compounds **8**–**24** are available from the authors.
