# Supplementary material for: Discovery of Cysteine and Its Derivatives as Novel Antiviral and Antifungal Agents
Source: Molecules. 2021 Jan 13;26(2):383. doi: 10.3390/molecules26020383 (PMC7828423; doi:10.3390/molecules26020383)
Supplement: Supplementary file 1 [file molecules-26-00383-s001.pdf]

molecules

MDPI

Article

Discovery of cysteine and its derivatives as novel antiviral and antifungal agents

Shan Yang [1,†], Tienan Wang[1,†], Yanan Zhou[1], Li Shi[1], Aidang Lu[1,*], Ziwen Wang[2,*]

[1] School of Chemical Engineering and Technology, Hebei University of Technology, Tianjin 300130, China; ys1826833034@qq.com (S. Y.); 2366463485@qq.com (T. W.); 1469585156@qq.com (Y. Z.); 2747831232@qq.com (L. S.); luaidang@hebut.edu.cn (A. L.)

[2] Tianjin Key Laboratory of Structure and Performance for Functional Molecules, College of Chemistry, Tianjin Normal University, Tianjin 300387, China; hxxywzw@tjnu.edu.cn (Z. W)

† These two authors contributed equally to this work.

* Correspondence: luaidang@hebut.edu.cn, Tel. 0086-22-60202812; hxxywzw@tjnu.edu.cn, Tel. 0086-22-23766531

Supplementary information

Contents

1 The detailed bio-assay procedures 3

1.1 Phytotoxic Activity 3

1.2 Antiviral Biological Assay 3

1.3 Detailed bioassay procedures for the fungicidal activities 4

2 Mode of Action Studies 5

3 ^{1}H, and ^{13}C NMR spectra of 8–24 6

^{1}H NMR and ^{13}C NMR spectra of **8** 6

^{1}H NMR and ^{13}C NMR spectra of **9** 7

^{1}H NMR and ^{13}C NMR spectra of **10** 8

^{1}H NMR and ^{13}C NMR spectra of **11** 9

^{1}H NMR and ^{13}C NMR spectra of **12** 10

^{1}H NMR and ^{13}C NMR spectra of **13** 11

^{1}H NMR and ^{13}C NMR spectra of **14** 12

^{1}H NMR and ^{13}C NMR spectra of **15** 13

^{1}H NMR and ^{13}C NMR spectra of **16** 14

^{1}H NMR and ^{13}C NMR spectra of **17** 15

^{1}H NMR and ^{13}C NMR spectra of **18** 16

^{1}H NMR and ^{13}C NMR spectra of **19** 17

^{1}H NMR and ^{13}C NMR spectra of **20** 18

^{1}H NMR and ^{13}C NMR spectra of **21** 19

^{1}H NMR and ^{13}C NMR spectra of **22** 20

^{1}H NMR and ^{13}C NMR spectra of **23** 21

^{1}H NMR and ^{13}C NMR spectra of **24** 22

1. The detailed bio-assay procedures

1.1. Phytotoxic Activity.

The growing 5–6 leaf stage tobaccos (*Nicotiana tabacum var Xanthi nc*) were selected. The compound solution (500 μg/mL) was smeared on the leaves and calculated the number of lesions after 0, 3, 7 and 10 days respectively. [1] There are three replicates for each compound.

1.2. Antiviral Biological Assay

1.2.1. Purification of Tobacco Mosaic Virus.

Using Gooding's method [2], the upper leaves of *Nicotiana tabacum* L. inoculated with TMV were selected and ground in phosphate buffer and then filtered through double-layer pledget. The filtrate was centrifuged at 10000 g, treated with PEG twice, and centrifuged again. The whole experiment was processed at 4 °C. Absorbance value was estimated at 260 nm by ultraviolet spectrophotometer

$$Virus\ concn = (A_{260} \times dilution\ ratio)\ E/{}_{1cm}^{0.1\%\ 260nm}$$

1.2.2. Antiviral Activity of Compounds against TMV in Vitro.

In vitro activity of the synthesized compounds against TMV was performed by the conventional half-leaf method. [1,3] Fresh leaf of the 5-6 growth stage of tobacco inoculated by the juice-leaf rubbing method (concentration of TMV is 5.88×10^{-2} μg/mL) was cut into halves along the main vein. The halves were immersed into the solution of different concentrations (see **Table 1**) of the compounds and double distilled water for 20 min, respectively, and then cultured at 25 °C for 72 h. Every concentration for each compound was replicated at least three times.

1.2.3. Protective Effect of Compounds against TMV in Vivo.

The compound solution was smeared on the left side and the solvent serving as control on the right side of growing *N. tabacum* L. leaves of the same ages. The leaves were then inoculated with the virus after 12 h. A brush was dipped in TMV of 6×10^{-3} mg/mL to inoculate the leaves, which were previously scattered with silicon carbide. The leaves were then washed with water and rubbed softly along the nervature once or twice. The local lesion numbers appearing 3–4 days after inoculation were counted. [1,4] There are three replicates for each compound.

1.2.4. Inactivation Effect of Compounds against TMV in Vivo.

The virus was inhibited by mixing with the compound solution at the same volume for 30 min. The mixture was then inoculated on the left side of the leaves of *N. tabacum* L., whereas the right side of the leaves was inoculated with the mixture of solvent and the virus for control. The local lesion numbers were recorded 3–4 days after inoculation. [1,4] There are three replicates for each compound.

1.2.5. Curative Effect of Compounds against TMV in Vivo.

Growing leaves of *N. tabacum* L. of the same ages were selected. TMV (concentration of 6.0×10^{-3} mg/mL) was dipped and inoculated on the whole leaves. Then the leaves were washed with water and dried. The compound solution was smeared on the left side, and the solvent was smeared on the right side for control. The local lesion numbers were then counted and recorded 3–4 days after inoculation. [1,4] There are three replicates for each compound. The in vitro and in vivo inhibition rates of the compound were then calculated according to the following formula ("av" means average, and controls were not treated with compound).

Inhibition rate (%) = [(av local lesion no. of control—av local lesion no. of drug-treated)/av local lesion no. of control] × 100%

1.3. Detailed bioassay procedures for the fungicidal activities

1.3.1. In Vitro Antifungal Bioassay.

The fungicidal activities of compounds were evaluated in mycelial growth tests conducted in artificial media against 14 plant pathogens at a rate of 50 *μ*g/mL. Each test compound was dissolved in a suitable amount of acetone and diluted with water containing 0.1% TW-80 to a concentration of 500 *μ*g/mL. To each petri dish was added 1 mL of the test solution and 9 mL of culture medium to make a 50 *μ*g/mL concentration of the test compound, while in another petri dish was added 1 mL distilled water containing 0.1% TW-80 and 9 mL of culture medium as a blank control. A 4 mm diameter of hyphal growth was cut using a hole puncher on a growing fungal culture and the hyphae were moved to the petri dish containing the test compound. Each assay was performed three times. The dishes were stored in controlled environment cabinets (24 ± 1 °C) for 4 days, after which the diameter of mycelial growth was measured and the percentage inhibition was calculated using the following equation: *Percentage inhibition (%)* = (*averaged diameter of mycelia in blank controls—averaged diameter of mycelia in medicated tablets*)/(*averaged diameter of mycelia in blank controls*—4 mm) × 100.[5]

1.3.2. In Vivo Antifungal Activity

The in vivo antifungal activity was evaluated by two methods. [5] In the two methods, azoxystrobin was evaluated as the positive controls at the same condition. Aqueous DMF（1%） containing 0.1% Tween 80 was set up as the blank control. Each tested compound was dissolved in DMF and then suspended in the distilled water (containing 0.1% Tween 80) to give the tested concentration of 200 $mg{\cdot}L^{-1}$. Three replicates were done for each compound. The inhibition percentage was expressed as the mean of values obtained in three independent experiments.

Method 1: Healthy tomato were sprayed with a solution of the tested compound at 200 $mg{\cdot}L^{-1}$ and evaporated under an ambient environment (~28 °C), which were washed and treated with 75% aqueous ethanol in advance. The epidermis of the fruits was punctured with an inoculating needle (*Ø*, 4.5 mm) and then the pathogen was inoculated with 4 mm plugs of corresponding pathogens. The fruits were assessed after cultivation at 25 °C for 4 days. The control efficacy of the target compounds was calculated as (*averaged diameter of mycelia in blank controls—averaged diameter of mycelia in medicated tablets*) / (*averaged diameter of mycelia in blank controls*—4 mm) × 100.

Method 2: Rape (*Sclerotinia sclerotiorum*) seedlings at the two-leaves stage were used to assay fungicidal activity. The tested compounds at the concentration of 200 $mg{\cdot}L^{-1}$ was sprayed on the surface of the rape leaves and evaporated under an ambient environment (~28 °C). After air drying, the rape leaves were inoculated by spraying the mycelial suspension of corresponding pathogens. Three days later, the symptoms were examined. The control efficacy of the target compounds was calculated as (*averaged disease index of blank controls—averaged disease index of drug treated*)/(*averaged disease index of blank controls*) × 100.

2. Mode of Action Studies

2.1. In vitro TMV rod assembly inhibition

TMV purification was performed according to the instructions by Leberman and Hao. [6,7] TMV RNA was purified by RNA pure virus kit (CoWin Biosciences) and TMV capsid protein (TMV CP) was isolated using glacial acetic acid as described by Fraenkel-Conrat. [8] Before assembly, 20S CP Disk was prepared by incubating 20 mg/mL TMV CP in 0.1 M phosphate buffer (pH 7.0) at 20 °C for 12 h. After incubation, in vitro TMV reconstitution reactions were performed by adding 5 μL of phosphate buffer (0.1 M, pH 7.0), 4 μL of 20S Disk (2 mg/mL) and 1 μL of TMV RNA (200 ng/μL). The assembly reaction mixture was incubated at 20 °C for 12 h and could be then transferred into the copper grid for transmission electron microscop (TEM) assay. The assembly reaction mixture (5 μL) was mixed with 5 μL 0.1 M phosphate buffer (pH 7.0) and dropped onto the copper film waiting for 5 minutes. After the incubation, the droplet was removed by filter paper and negatively stained by 2% phosphotungstic acid (pH 7.0) for three minutes. After removing the staining agent, the copper

was placed at 37 ℃ for 2 h for drying. The morphology of the reconstituted TMV rods was imaged at 200 keV on a CCD camera. For the inhibition tests with the compounds, in vitro TMV reconstitution inhibition reactions were performed by adding 4.8 μL of phosphate buffer (0.1 M, pH 7.0), 4 μL of 20S Disk (2 mg/mL), 1 μL of TMV RNA (200 ng/μL) and 0.2 μL of DMSO or the compound (10 μM). All treatments were repeated over time to validate the results. [6]

2.2. In vitro 20S CP Disk assembly inhibition

For the inhibition tests with the compounds, TMV CP was first adjusted to 20.1 mg/mL with 0.1 M phosphate buffer (pH 7.0). In vitro 20S CP Disk assembly reactions were performed by adding 9.8 μL TMV CP (20.1 mg/mL) and 0.2 μL DMSO or the compound (10 μM). The assembly reaction was incubated at 20 °C for 12 h. The morphology of the 20S CP Disk was imaged via TEM at 200 keV on a CCD camera. All treatments were repeated over time for confirmation. [6]

References

1. Wang, Z. W.; Wei, P.; Wang, L. Z.; Wang, Q. M. Design, synthesis, and anti-tobacco mosaic virus (TMV) activity of phenanthroindolizidines and their analogues. *J. Agric. Food Chem*. **2012**, *60*, 10212–10219.
2. Gooding, G. V., Jr.; Hebert, T. T. A simple technique for purification of tobacco mosaic virus in large quantities. *Phytopathology* **1967**, 57, 1285–1290.
3. Chen, N. C. Pesticide’s Bioassay Technique; Beijing Agriculture University: Beijing, People's Republic of China, **1990**; p 194.
4. Li, S. Z.; Wang, D. M.; Jiao, S. M. In Pesticide Experiment Methods-Fungicide Sector; Li, S. Z., Ed.; Agriculture Press of China: Beijing, China, **1991**; 93–94.
5. Zhao, H. P.; Liu, Y. X.; Cui, Z. P.; Beattie, D.; Gu, Y. C.; Wang, Q. M. Design, synthesis, and biological activities of arylmethylamine substituted chlorotriazine and methylthiotriazine compounds. *J. Agric. Food Chem.* **2011**, *59*, 11711–11717.
6. Hao, Y. N.; Wang, K. H.; Wang, Z. W.; Liu, Y. X.; Ma, D. Y.; Wang, Q. M. Luotonin A and its derivatives as novel antiviral and antiphytopathogenic fungus agents. *J. Agric. Food Chem.* **2019**, *68*, 8764–8773.
7. Leberman, R. Isolation of plant viruses by means of simple coacervates. *Virol*. **1966**, 30, 341–347.
8. Fraenkel Conrat, H.; Williams, R. C. Reconstitution of active tobacco mosaic virus fromits inactive protein and nucleic acid components. *Proc. Natl. Acad. Sci. USA* **1955**, *41*, 690–698.

3 ^{1}H, and ^{13}C NMR spectra of 8–24

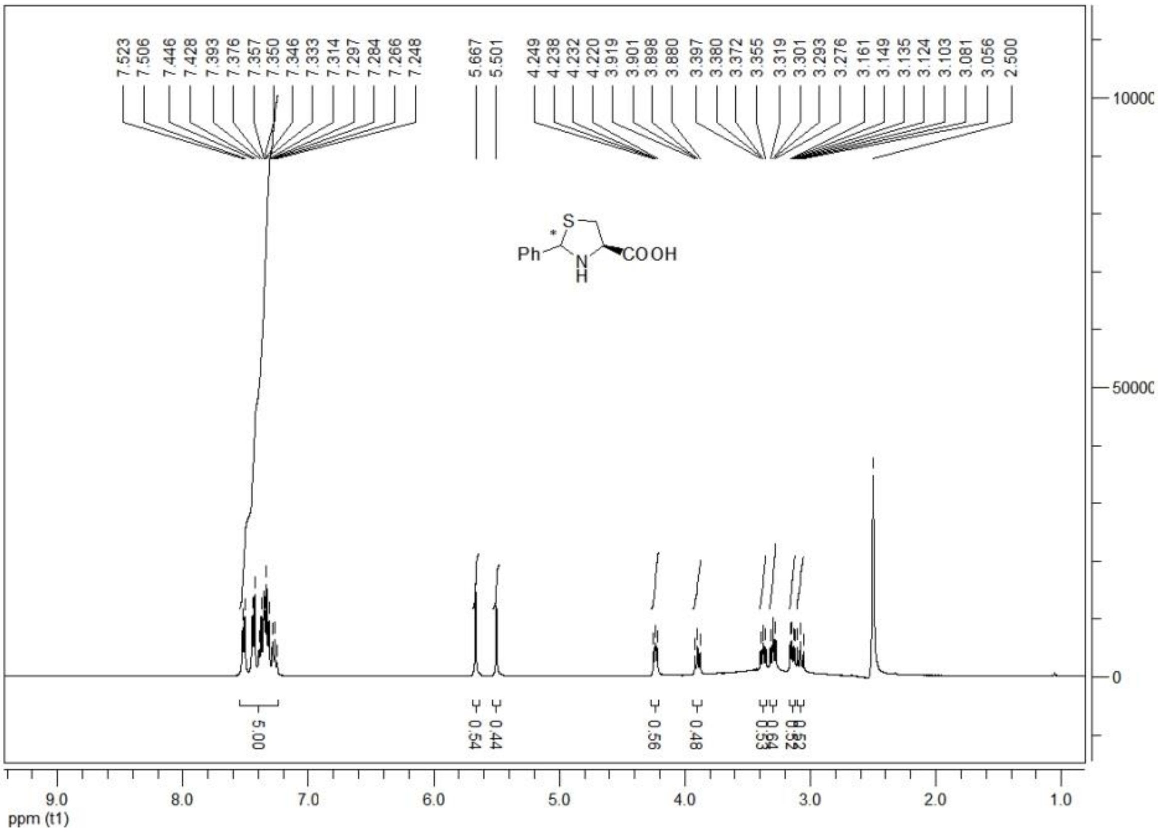

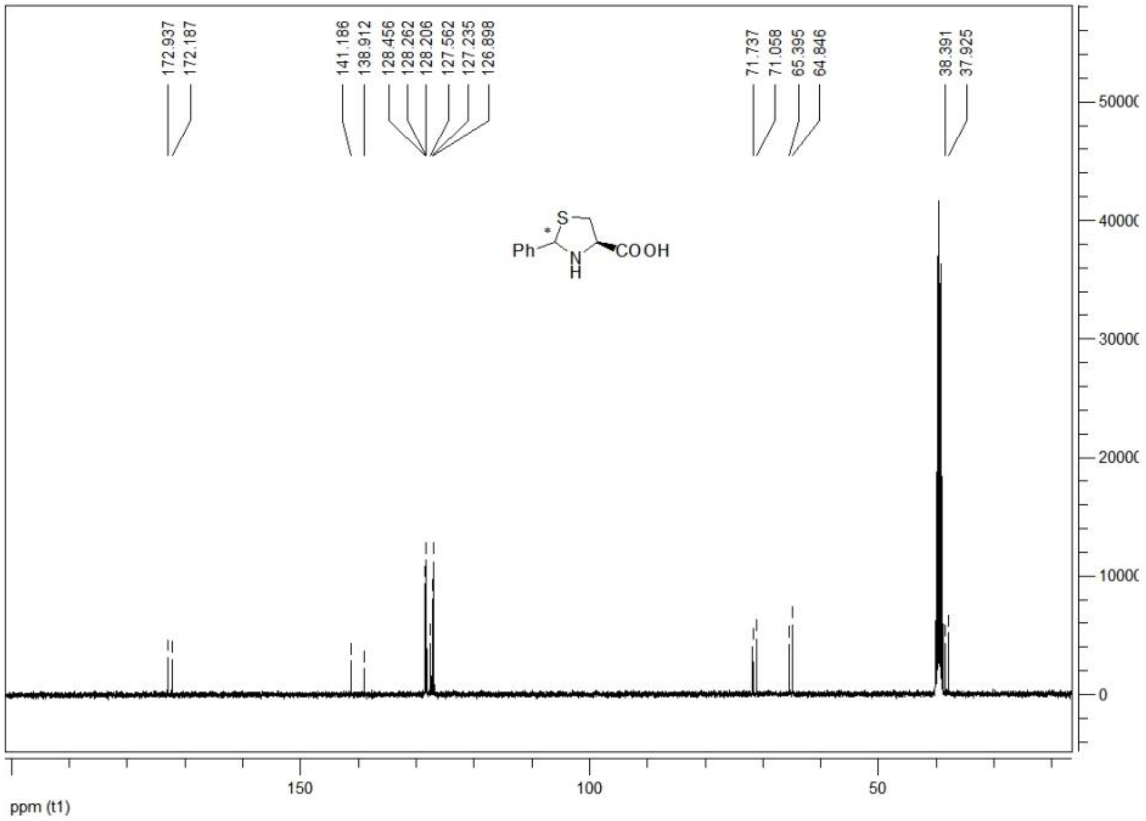

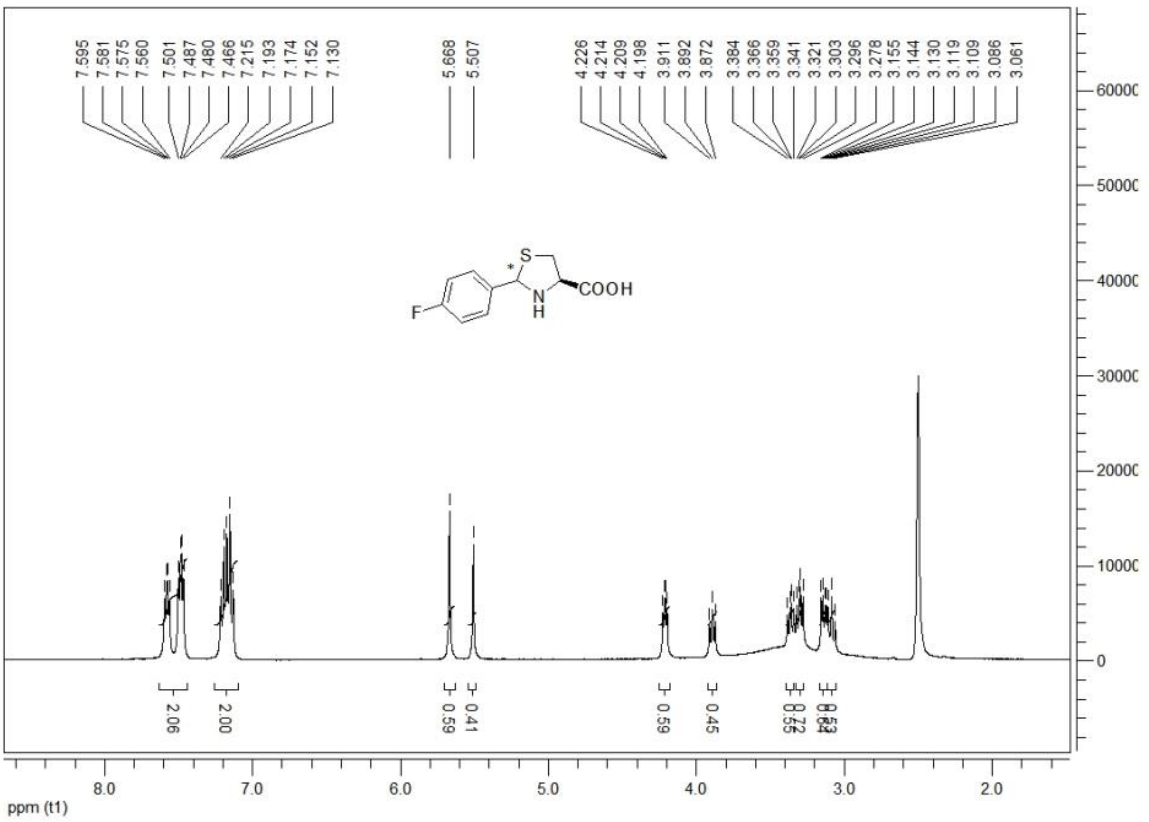
7.595
7.581
7.575
7.560
7.501
7.487
7.480
7.466
7.215
7.193
7.174
7.152
7.130
5.668
5.507
4.226
4.214
4.209
4.198
3.911
3.892
3.872
3.384
3.366
3.359
3.341
3.321
3.303
3.296
3.278
3.155
3.144
3.130
3.119
3.109
3.086
3.061
S
N
H
COOH
F
*
2.06
2.00
0.59
0.41
0.59
0.45
8.0
7.0
6.0
5.0
4.0
3.0
2.0
ppm (t1)

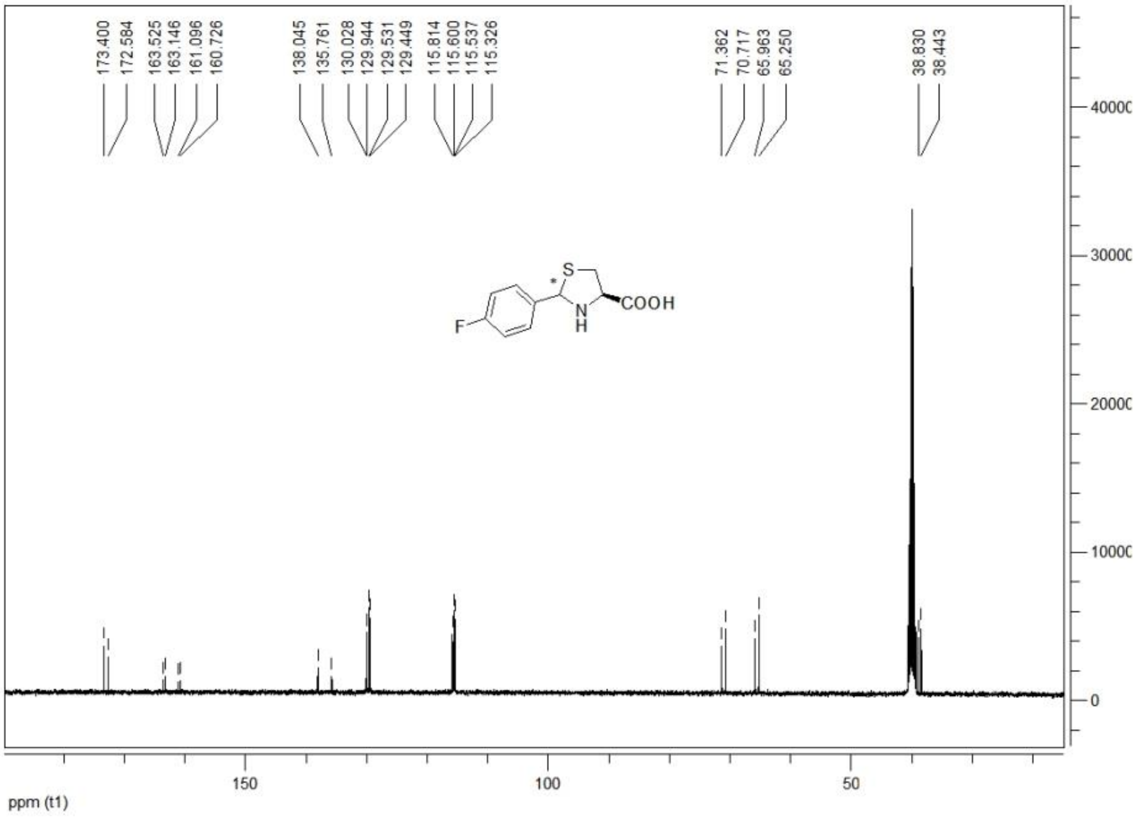
173.400
172.584
163.525
163.146
161.096
160.726
138.045
135.761
130.028
129.944
129.531
129.449
115.814
115.600
115.537
115.326
71.362
70.717
65.963
65.250
38.830
38.443
S
N
H
COOH
F
*
150
100
50
ppm (t1)

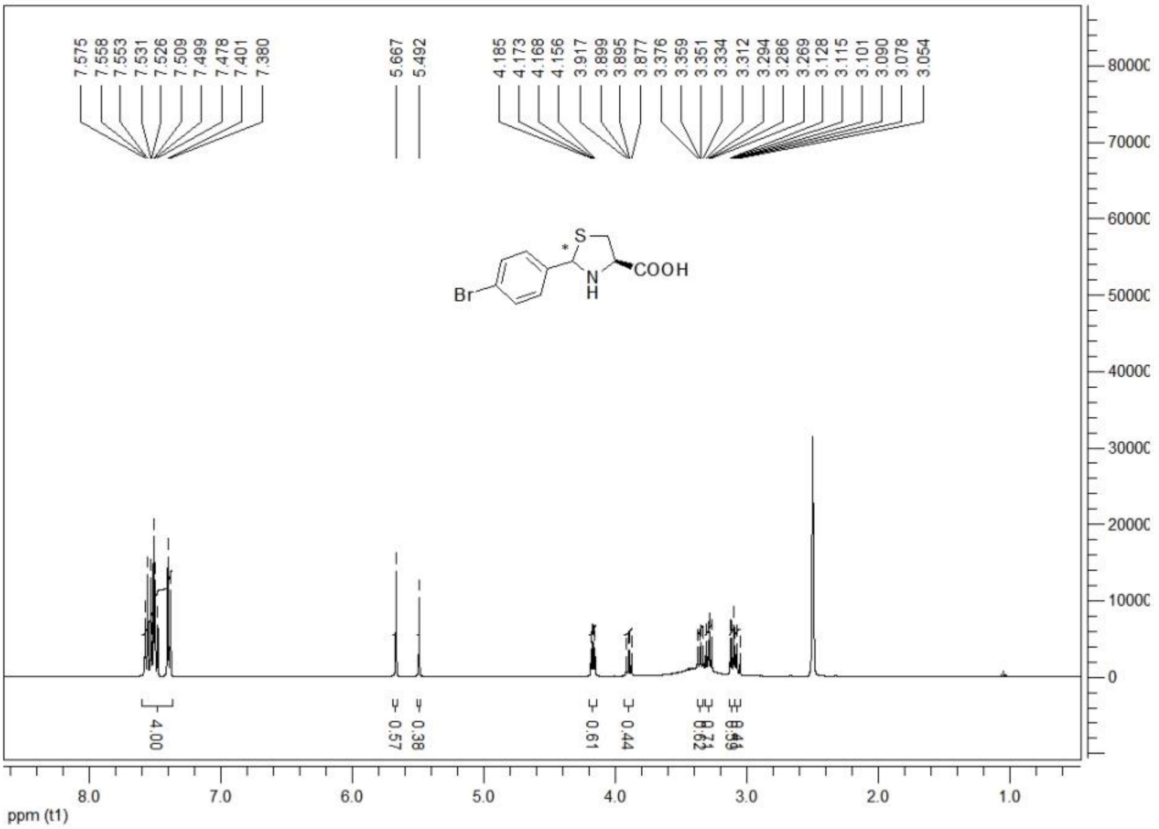
7.575
7.558
7.553
7.531
7.526
7.509
7.499
7.478
7.401
7.380
5.667
5.492
4.185
4.173
4.168
4.156
3.917
3.899
3.895
3.877
3.376
3.359
3.351
3.334
3.312
3.294
3.286
3.269
3.128
3.115
3.101
3.090
3.078
3.054
S
N
H
COOH
Br
4.00
0.57
0.38
0.61
0.44
8.0
7.0
6.0
5.0
4.0
3.0
2.0
1.0
ppm (t1)

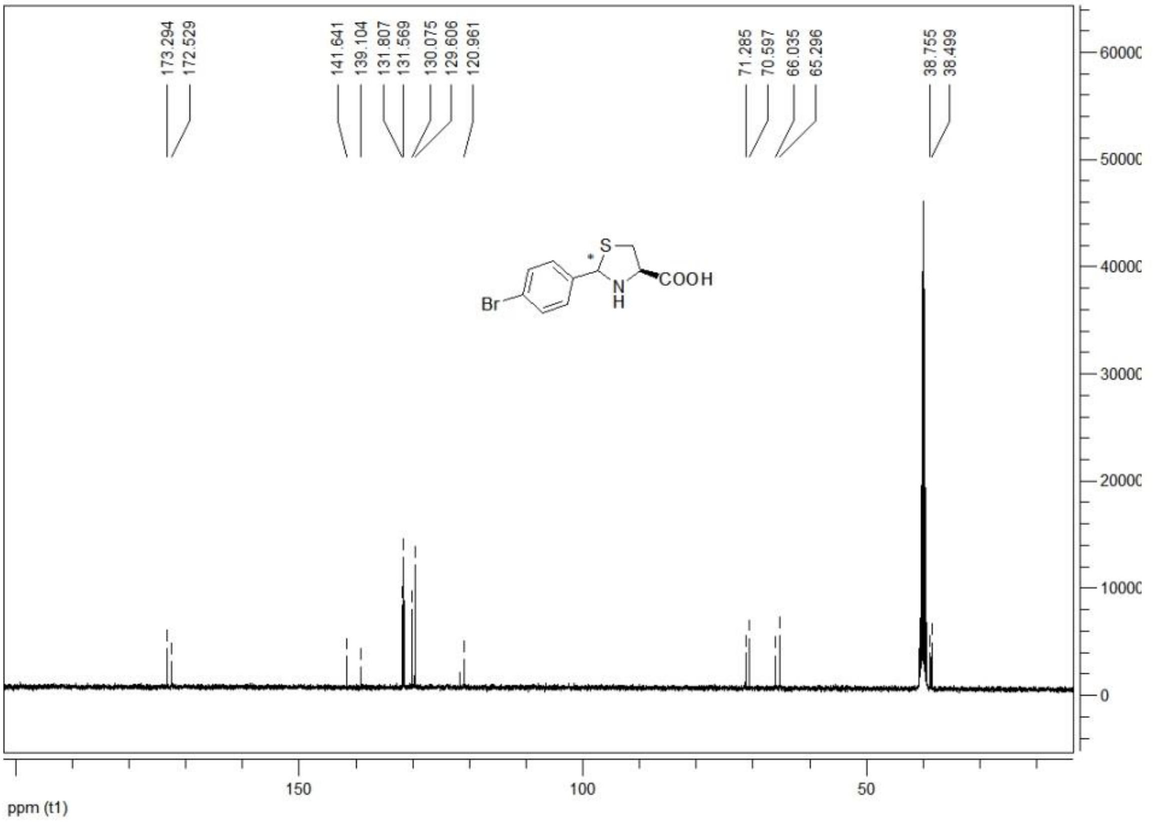
173.294
172.529
141.641
139.104
131.807
131.569
130.075
129.606
120.961
71.285
70.597
66.035
65.296
38.755
38.499
S
N
H
COOH
Br
150
100
50
ppm (t1)

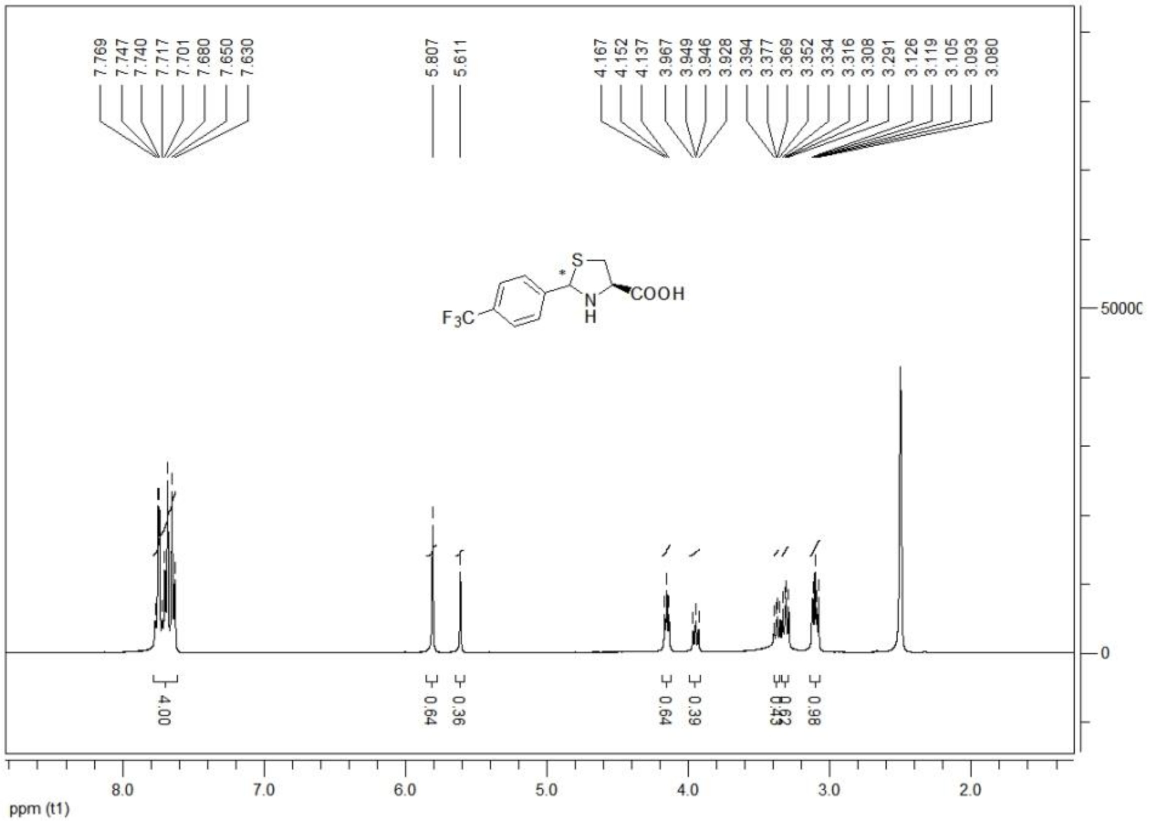
7.769
7.747
7.740
7.717
7.701
7.680
7.650
7.630
5.807
5.611
4.167
4.152
4.137
3.967
3.949
3.946
3.928
3.394
3.377
3.369
3.352
3.334
3.316
3.308
3.291
3.126
3.119
3.105
3.093
3.080
F3C
S
N
H
COOH
50000
0
4.00
0.64
0.36
0.64
0.39
0.98
8.0
7.0
6.0
5.0
4.0
3.0
2.0
ppm (t1)

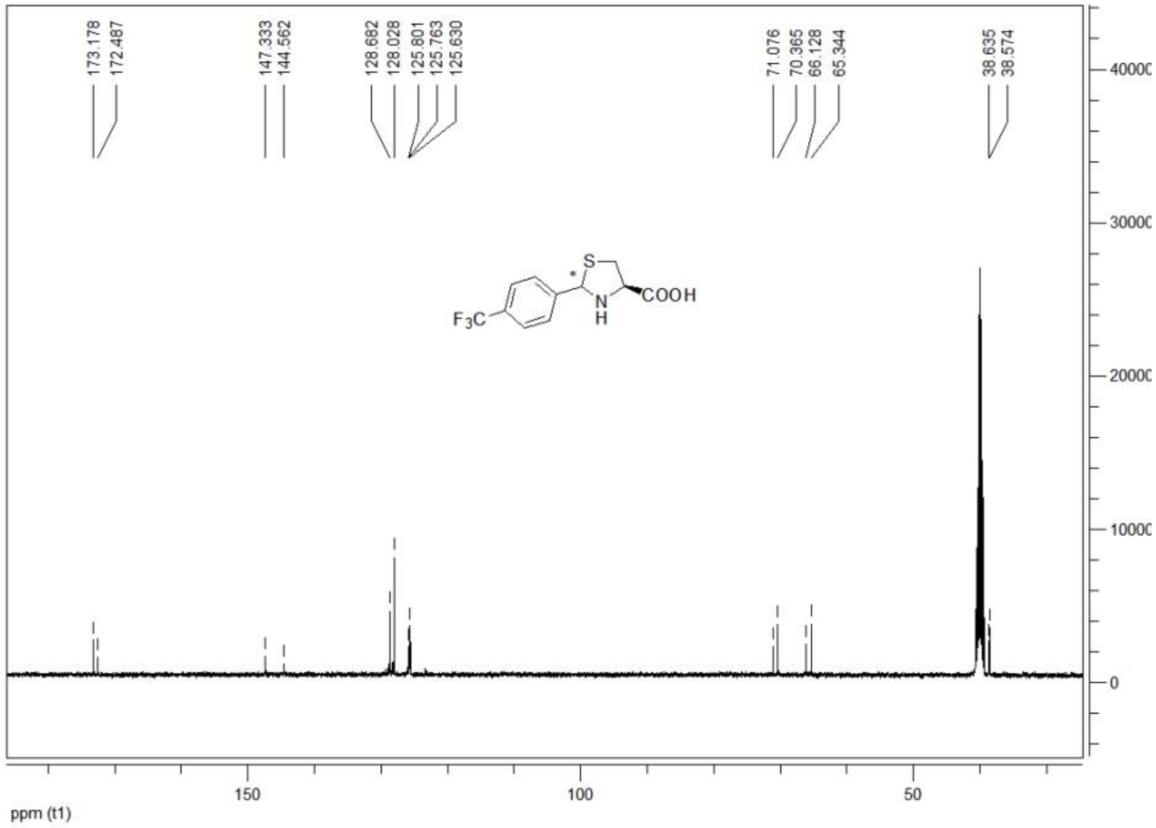
173.178
172.487
147.333
144.562
128.682
128.028
125.801
125.763
125.630
71.076
70.365
66.128
65.344
38.635
38.574
F3C
S
N
H
COOH
40000
30000
20000
10000
0
150
100
50
ppm (t1)

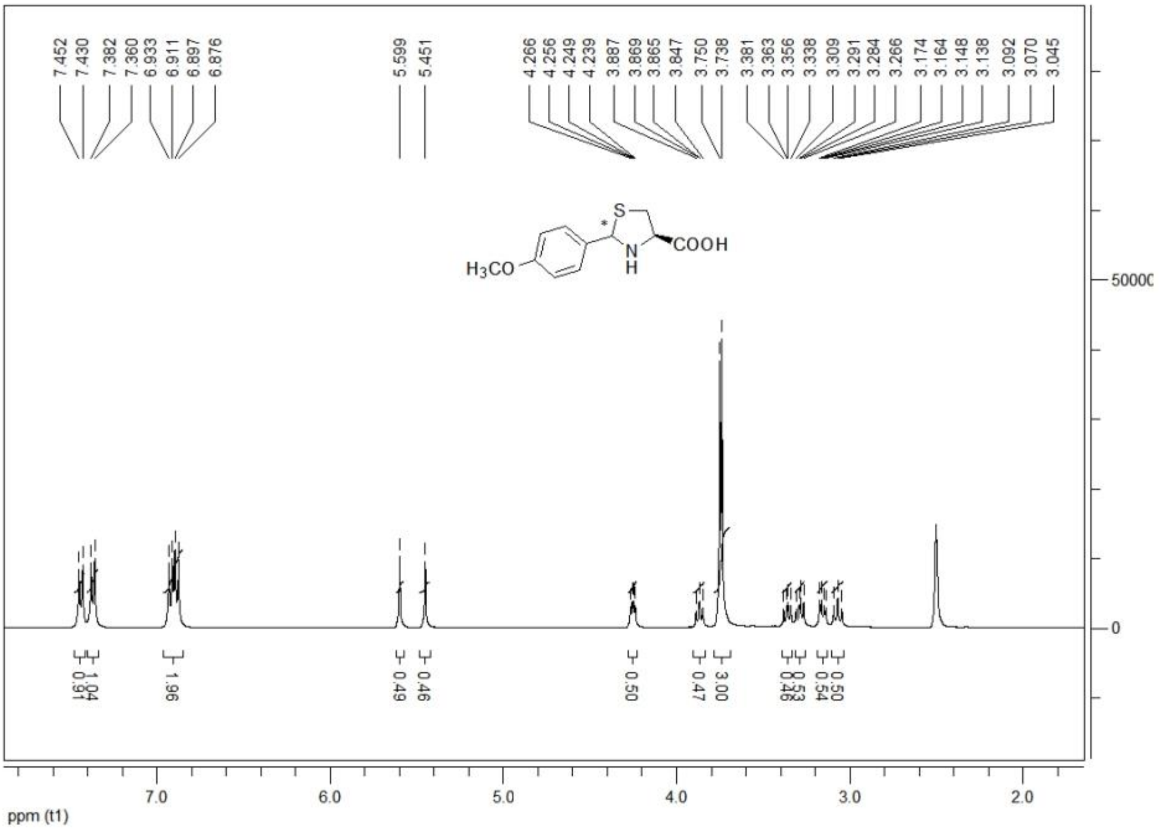
7.452
7.430
7.382
7.360
6.933
6.911
6.897
6.876
5.599
5.451
4.266
4.256
4.249
4.239
3.887
3.869
3.865
3.847
3.750
3.738
3.381
3.363
3.356
3.338
3.309
3.291
3.284
3.266
3.174
3.164
3.148
3.138
3.092
3.070
3.045
H_3CO
S
N
H
*
COOH
50000
0
0.91
1.04
1.96
0.49
0.46
0.50
0.47
3.00
0.48
0.53
0.54
0.50
7.0
6.0
5.0
4.0
3.0
2.0
ppm (t1)

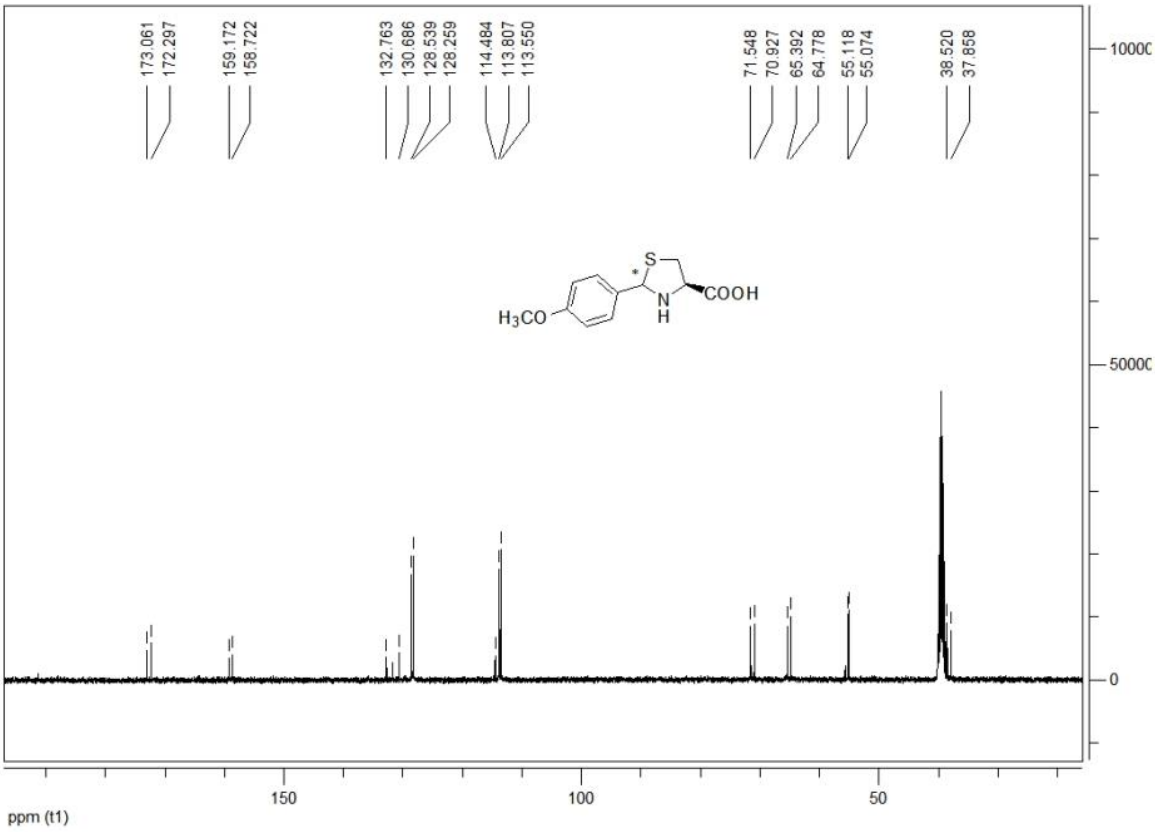
173.061
172.297
159.172
158.722
132.763
130.686
128.539
128.259
114.484
113.807
113.550
71.548
70.927
65.392
64.778
55.118
55.074
38.520
37.858
H_3CO
S
N
H
*
COOH
100000
50000
0
150
100
50
ppm (t1)

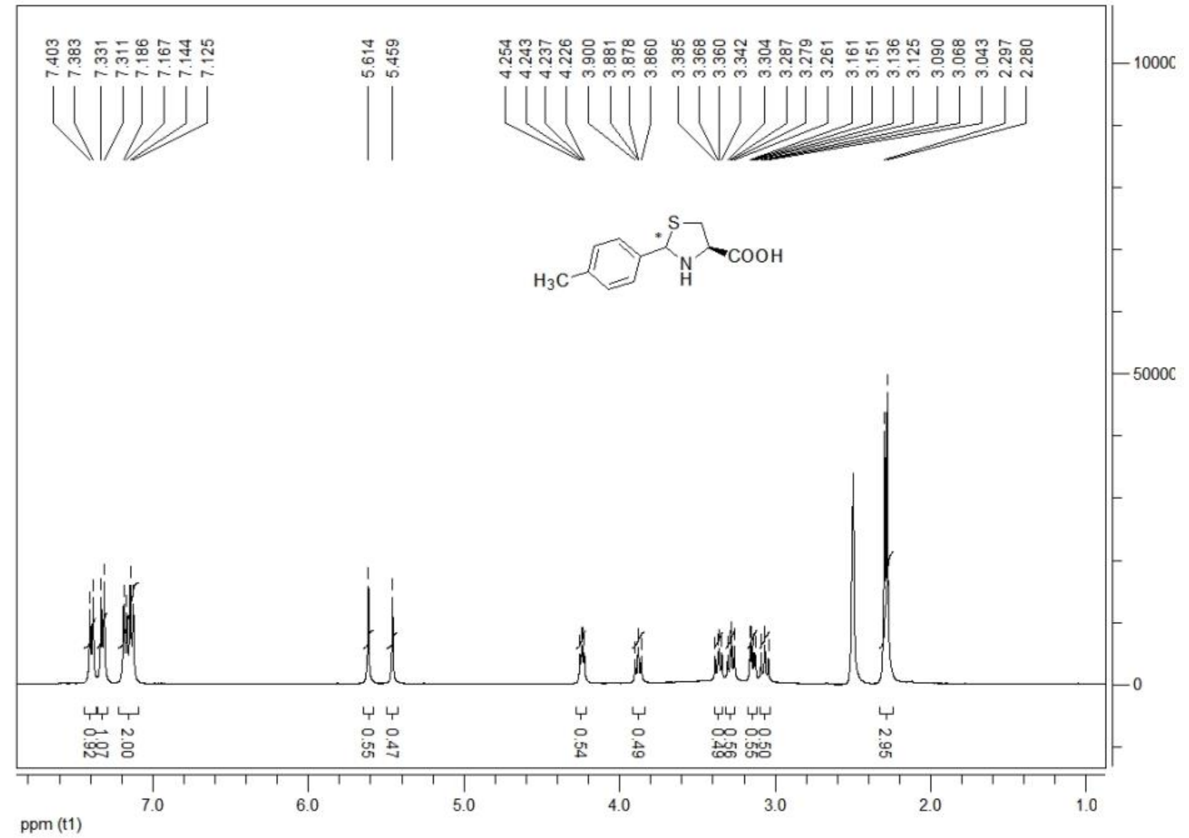
7.403
7.383
7.331
7.311
7.186
7.167
7.144
7.125
5.614
5.459
4.254
4.243
4.237
4.226
3.900
3.881
3.878
3.860
3.385
3.368
3.360
3.342
3.304
3.287
3.279
3.261
3.161
3.151
3.136
3.125
3.090
3.068
3.043
2.297
2.280
COOH
H_3C
S
N
H
0.92
1.07
2.00
0.55
0.47
0.54
0.49
2.95
7.0
6.0
5.0
4.0
3.0
2.0
1.0
ppm (t1)
10000
50000
0

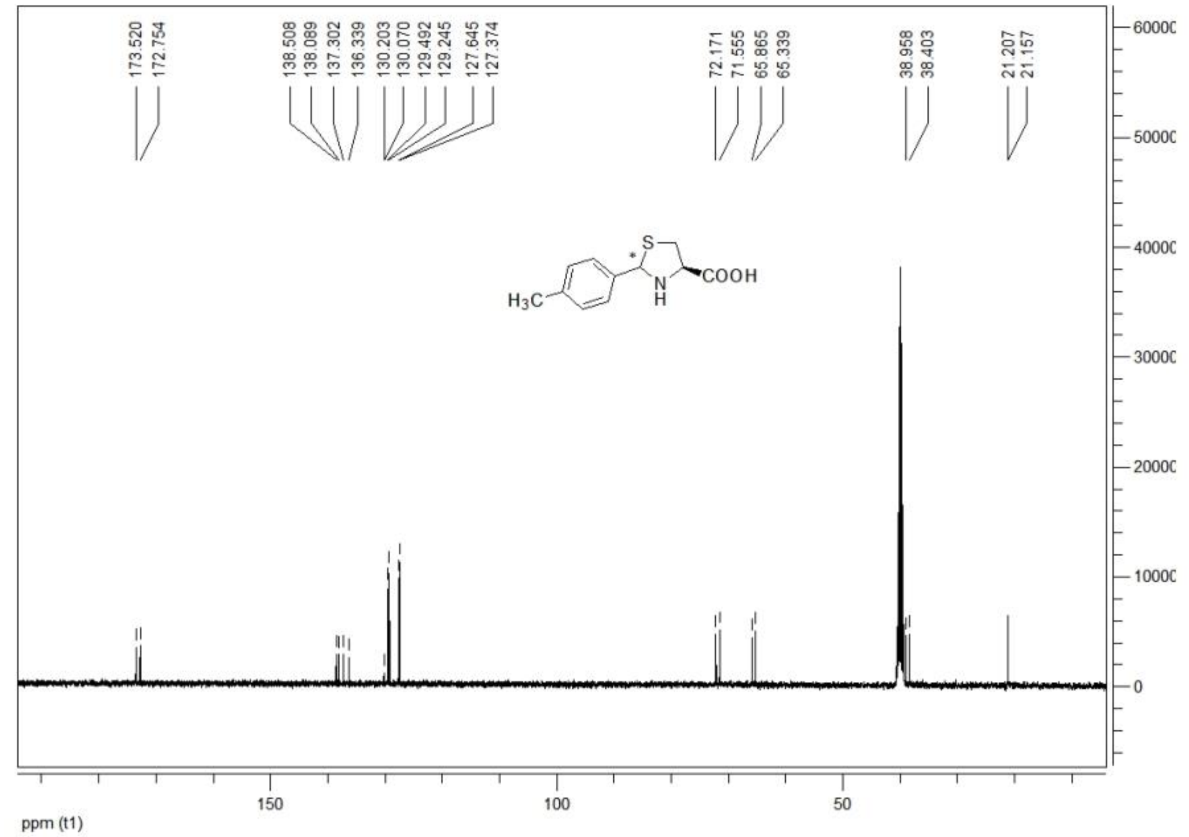
173.520
172.754
138.508
138.089
137.302
136.339
130.203
130.070
129.492
129.245
127.645
127.374
72.171
71.555
65.865
65.339
38.958
38.403
21.207
21.157
COOH
H_3C
S
N
H
150
100
50
ppm (t1)
60000
50000
40000
30000
20000
10000
0

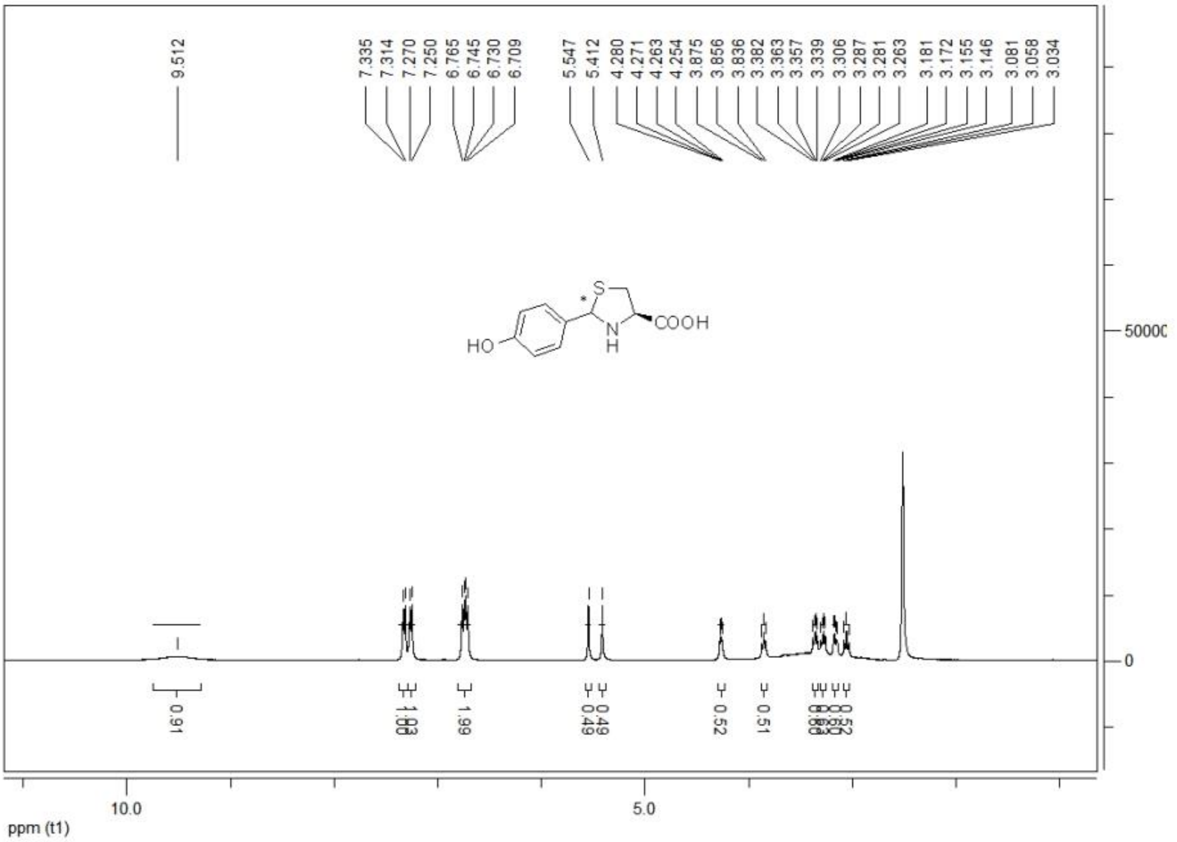
9.512
7.335
7.314
7.270
7.250
6.765
6.745
6.730
6.709
5.547
5.412
4.280
4.271
4.263
4.254
3.875
3.856
3.836
3.382
3.363
3.357
3.339
3.306
3.287
3.281
3.263
3.181
3.172
3.155
3.146
3.081
3.058
3.034
HO
S
N
H
COOH
*
50000
0
0.91
1.99
0.49
0.49
0.52
0.51
10.0
5.0
ppm (t1)

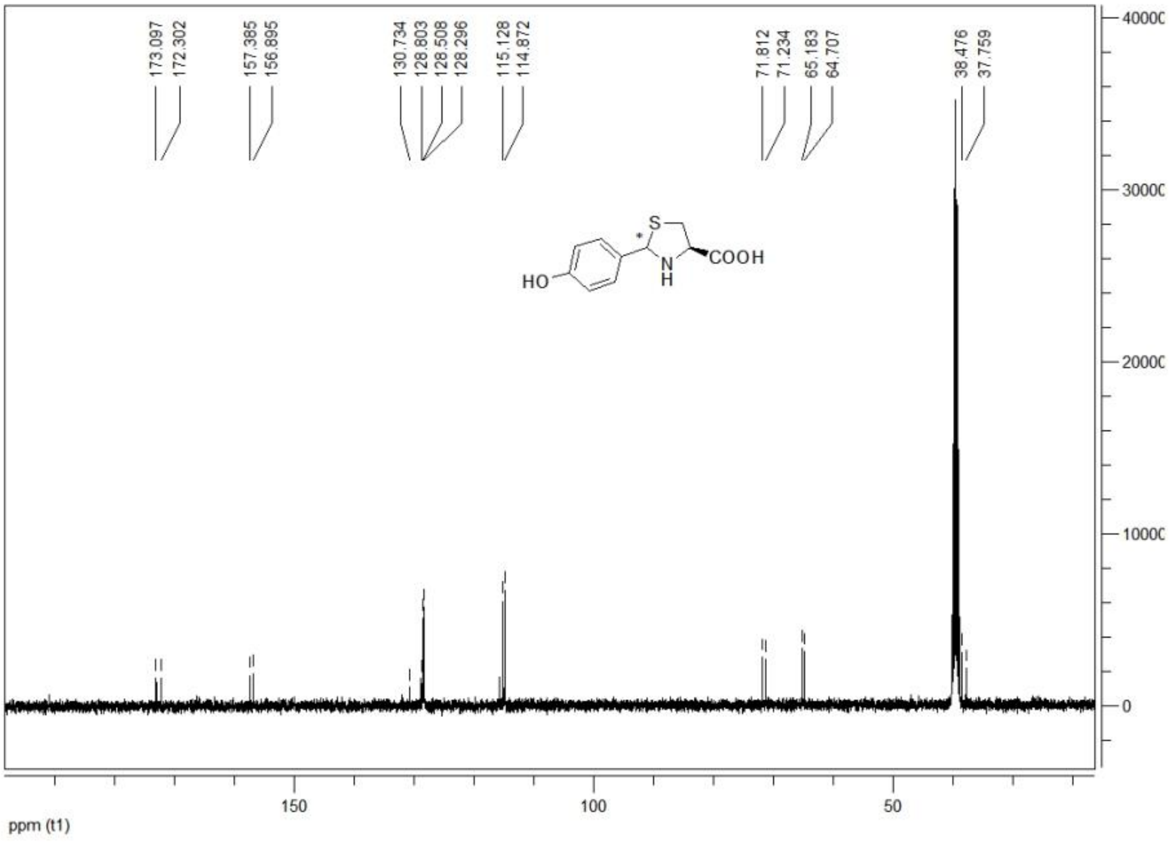
173.097
172.302
157.385
156.895
130.734
128.803
128.508
128.296
115.128
114.872
71.812
71.234
65.183
64.707
38.476
37.759
HO
S
N
H
COOH
*
40000
30000
20000
10000
0
150
100
50
ppm (t1)

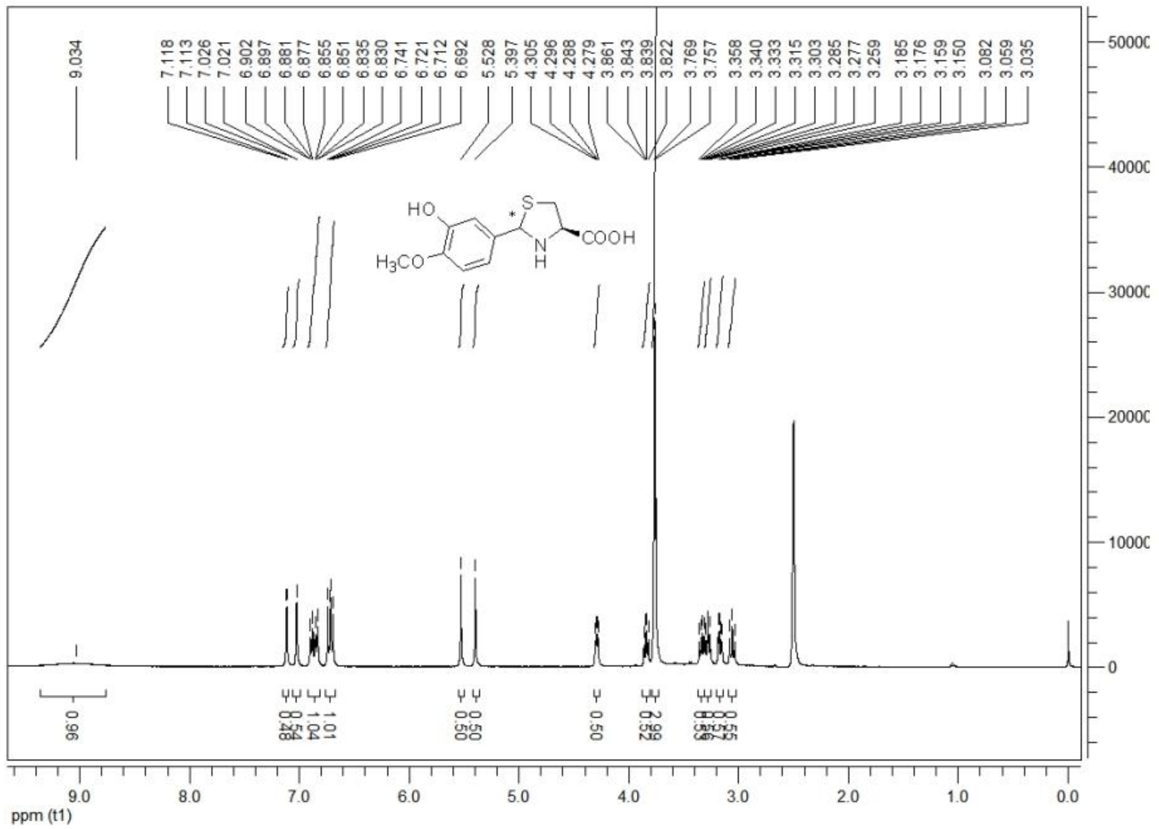
9.034
7.118
7.113
7.026
7.021
6.902
6.897
6.881
6.877
6.855
6.851
6.835
6.830
6.741
6.721
6.712
6.692
5.528
5.397
4.305
4.296
4.288
4.279
3.861
3.843
3.839
3.822
3.769
3.757
3.358
3.340
3.333
3.315
3.303
3.285
3.277
3.259
3.185
3.176
3.159
3.150
3.082
3.059
3.035
HO
H3CO
S
N
H
COOH
*
0.96
1.01
1.04
0.50
0.50
0.50
2.99
ppm (t1)

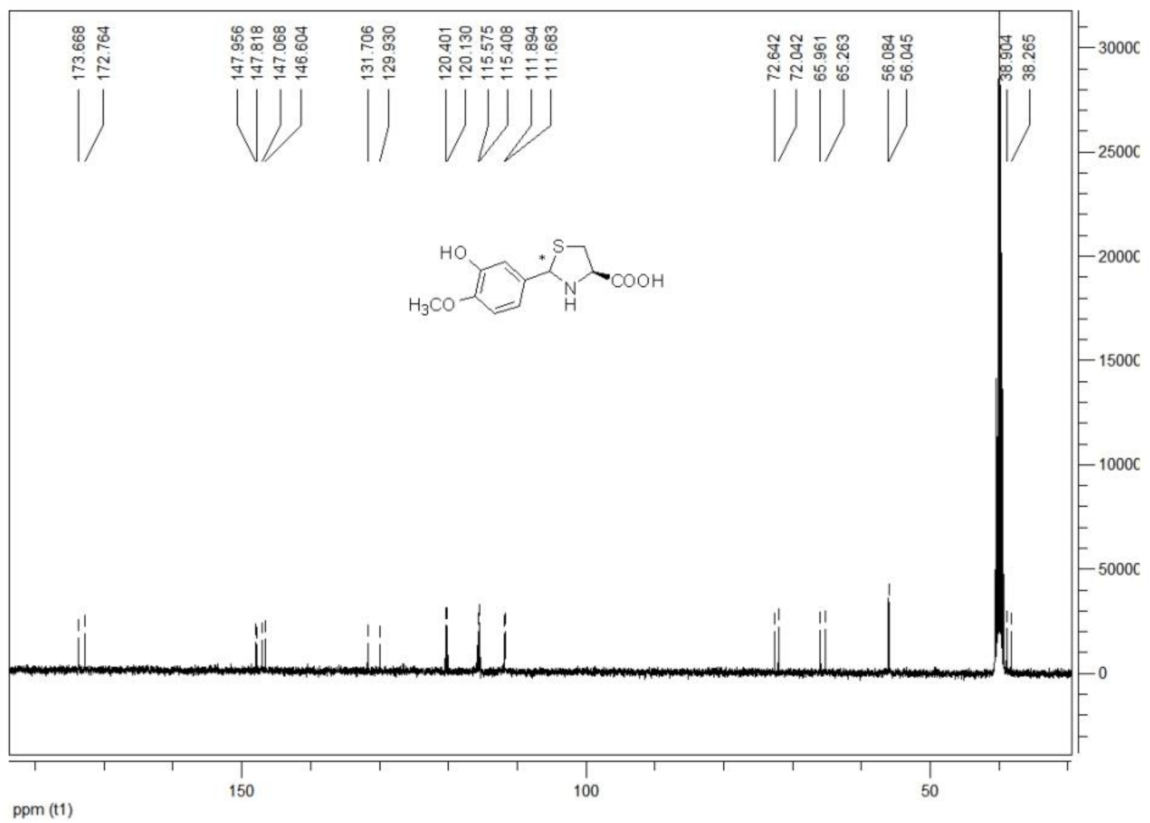
173.668
172.764
147.956
147.818
147.068
146.604
131.706
129.930
120.401
120.130
115.575
115.408
111.894
111.683
72.642
72.042
65.961
65.263
56.084
56.045
38.904
38.265
HO
H3CO
S
N
H
COOH
*
ppm (t1)

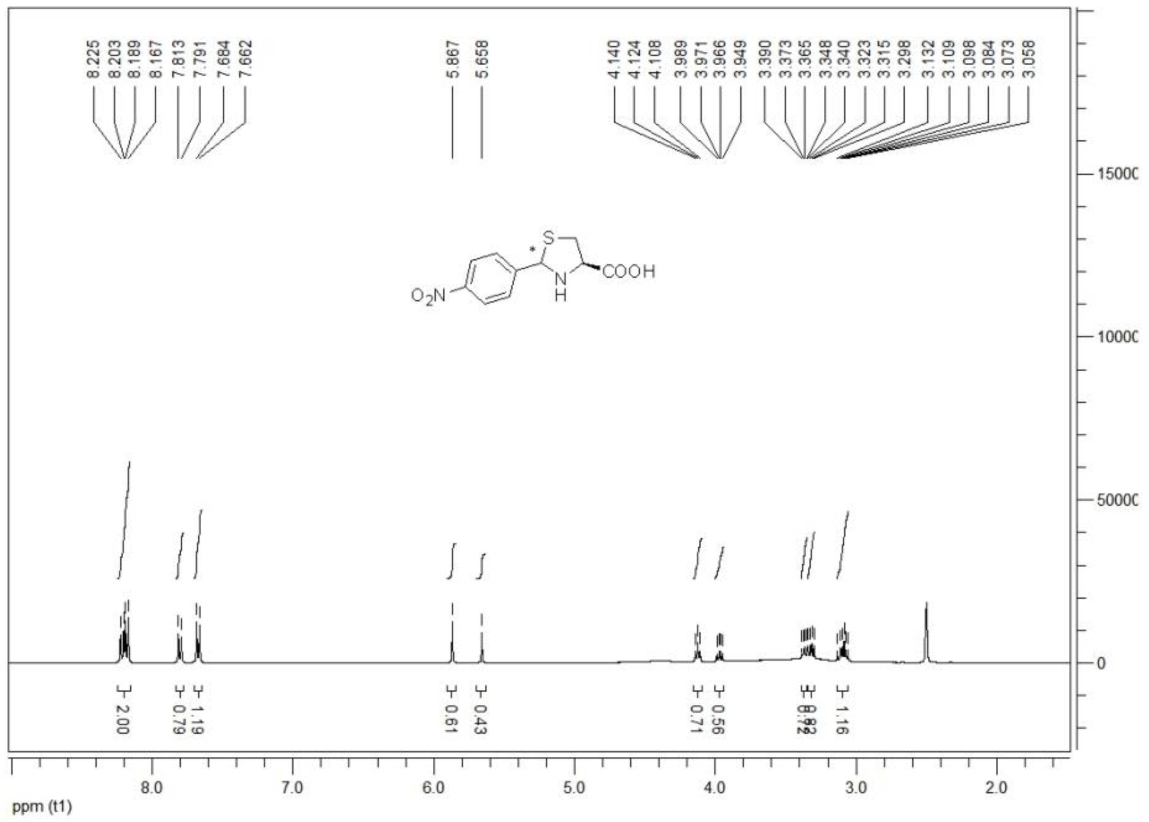
8.225
8.203
8.189
8.167
7.813
7.791
7.684
7.662
5.867
5.658
4.140
4.124
4.108
3.989
3.971
3.966
3.949
3.390
3.373
3.365
3.348
3.340
3.323
3.315
3.298
3.132
3.109
3.098
3.084
3.073
3.058
COOH
2.00
0.79
1.19
0.61
0.43
0.71
0.56
1.16
ppm (t1)

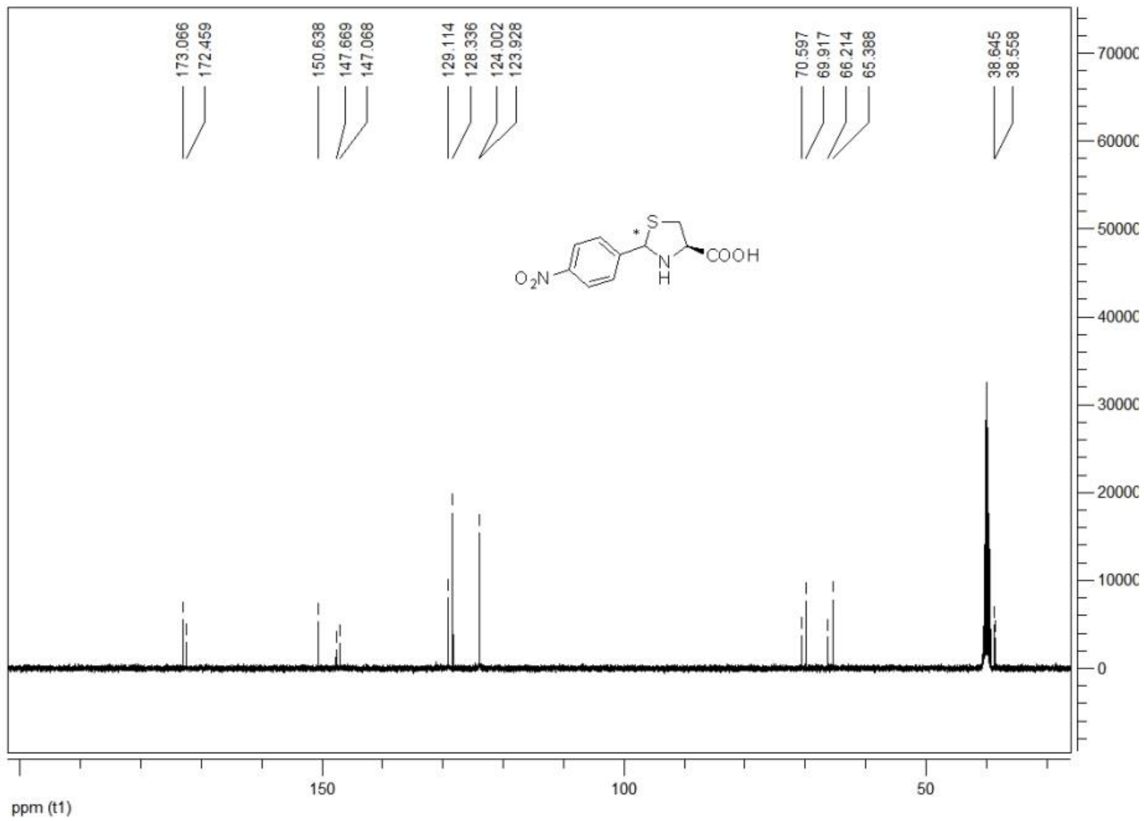
173.066
172.459
150.638
147.669
147.068
129.114
128.336
124.002
123.928
70.597
69.917
66.214
65.388
38.645
38.558
COOH
ppm (t1)

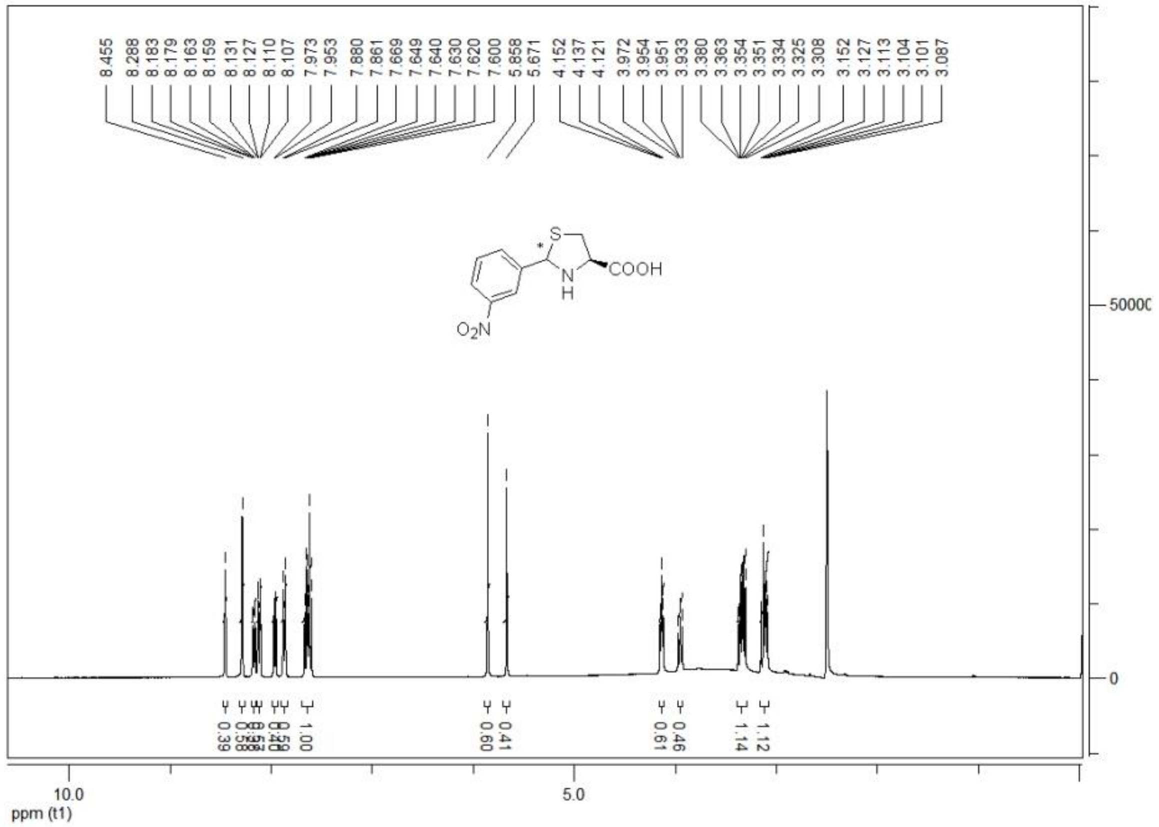
8.455
8.288
8.183
8.179
8.163
8.159
8.131
8.127
8.110
8.107
7.973
7.953
7.880
7.861
7.669
7.649
7.640
7.630
7.620
7.600
5.858
5.671
4.152
4.137
4.121
3.972
3.954
3.951
3.933
3.380
3.363
3.354
3.351
3.334
3.325
3.308
3.152
3.127
3.113
3.104
3.101
3.087
S
N
H
COOH
O_2N
50000
0
0.39
1.00
0.60
0.41
0.61
0.46
1.14
1.12
10.0
5.0
ppm (t1)

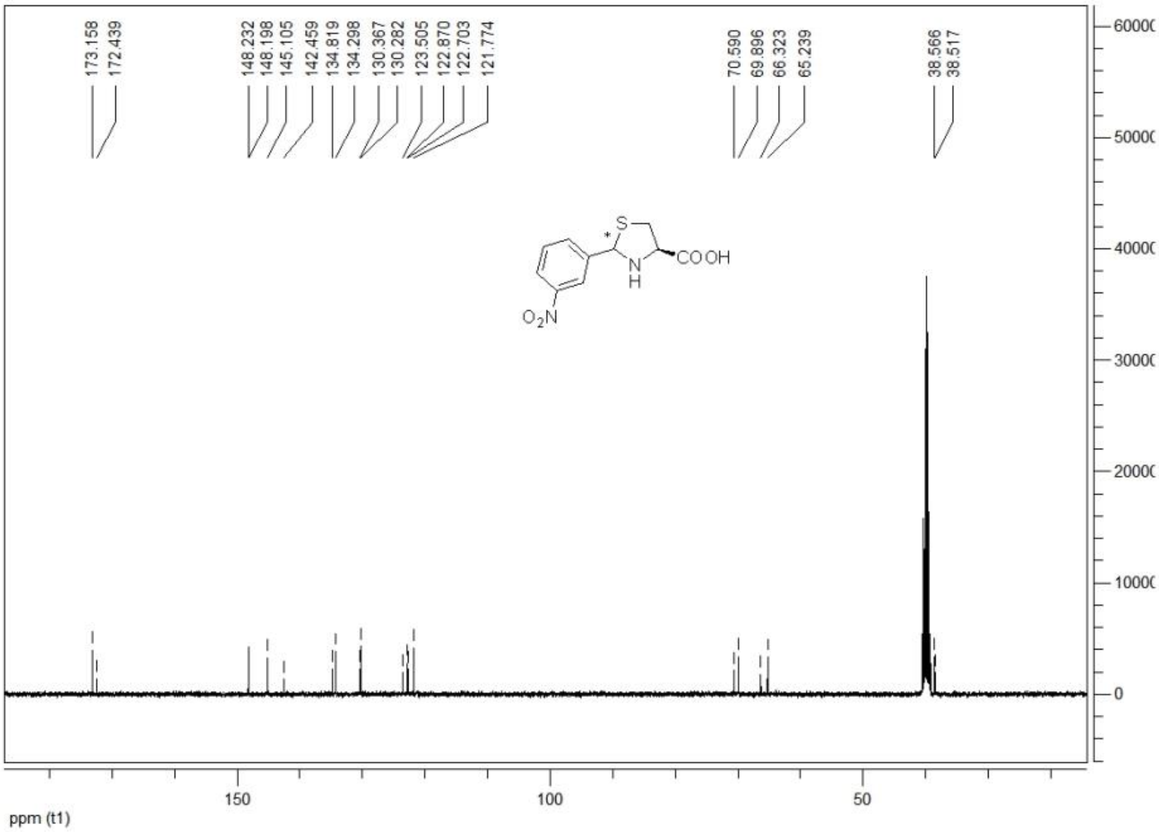
173.158
172.439
148.232
148.198
145.105
142.459
134.819
134.298
130.367
130.282
123.505
122.870
122.703
121.774
70.590
69.896
66.323
65.239
38.566
38.517
S
N
H
COOH
O_2N
60000
50000
40000
30000
20000
10000
0
150
100
50
ppm (t1)

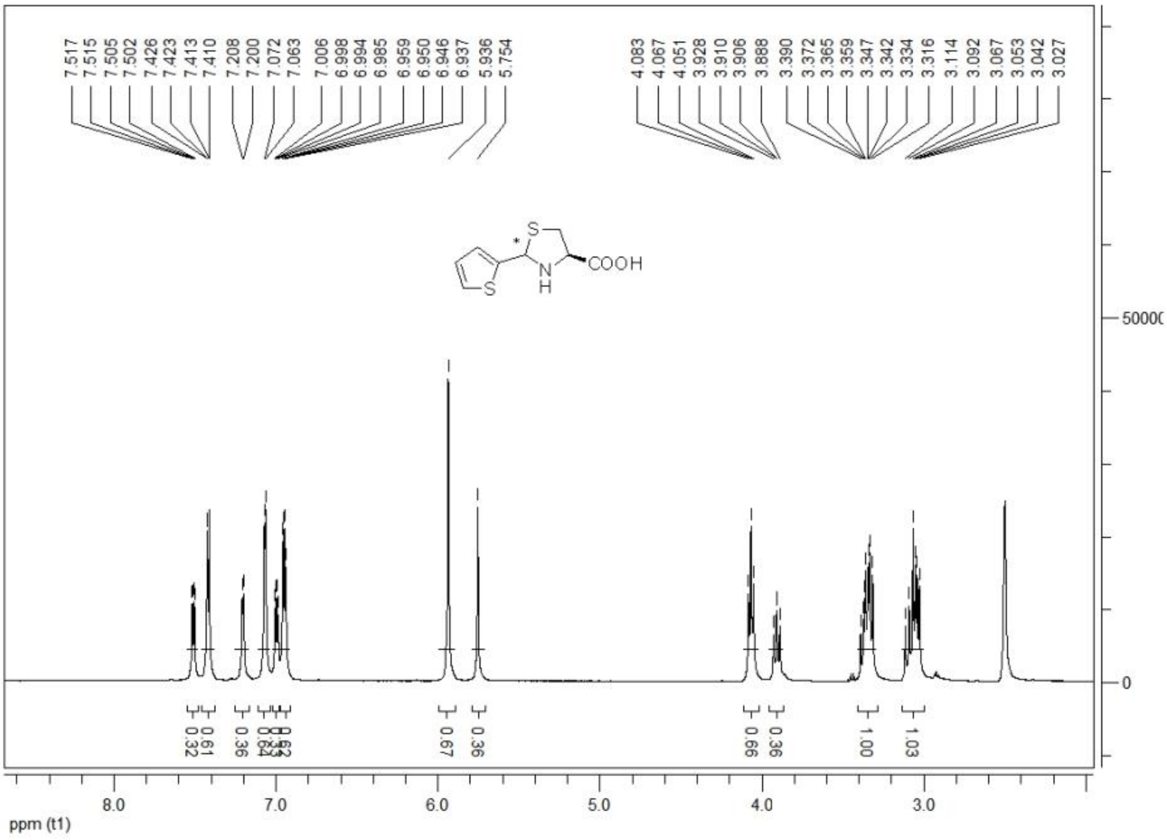
7.517
7.515
7.505
7.502
7.426
7.423
7.413
7.410
7.208
7.200
7.072
7.063
7.006
6.998
6.994
6.985
6.959
6.950
6.946
6.937
5.936
5.754
4.083
4.067
4.051
3.928
3.910
3.906
3.888
3.390
3.372
3.365
3.359
3.347
3.342
3.334
3.316
3.114
3.092
3.067
3.053
3.042
3.027
S
N
H
COOH
S
*
50000
0
0.32
0.61
0.36
0.67
0.36
0.66
0.36
1.00
1.03
8.0
7.0
6.0
5.0
4.0
3.0
ppm (t1)

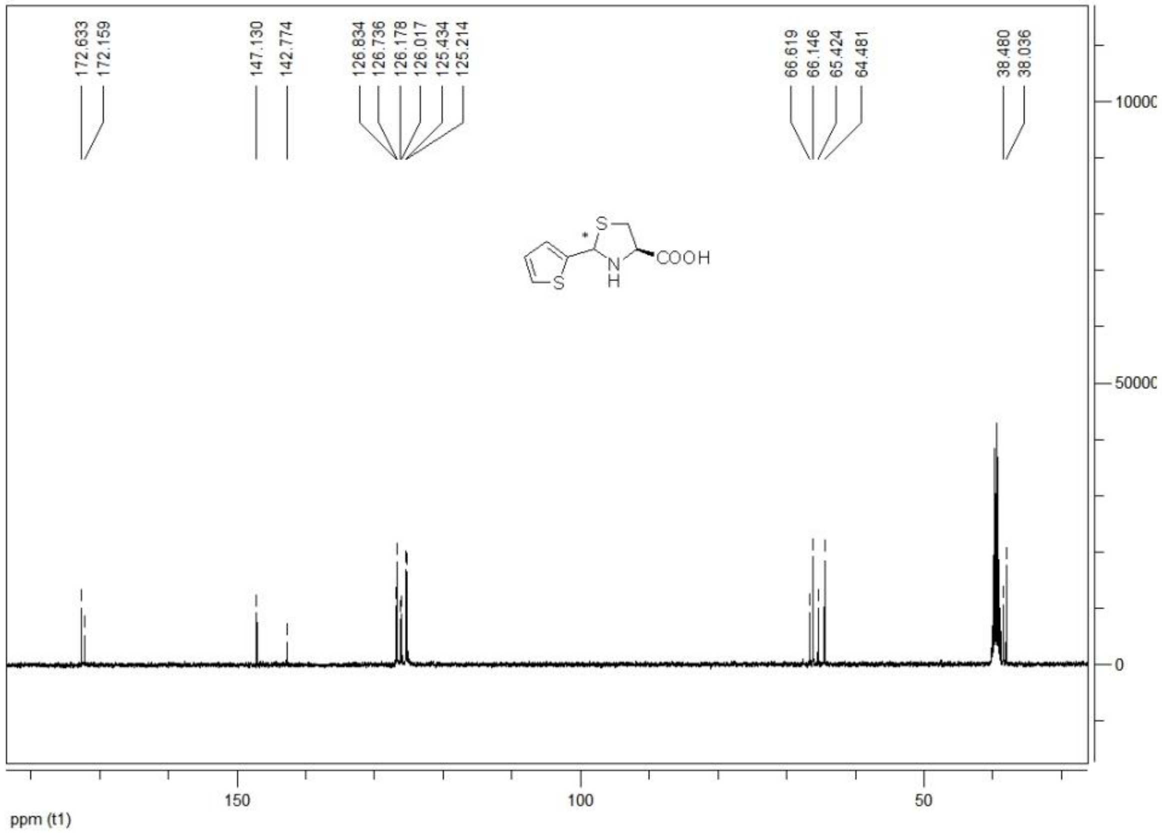
172.633
172.159
147.130
142.774
126.834
126.736
126.178
126.017
125.434
125.214
66.619
66.146
65.424
64.481
38.480
38.036
S
N
H
COOH
S
*
10000
50000
0
150
100
50
ppm (t1)

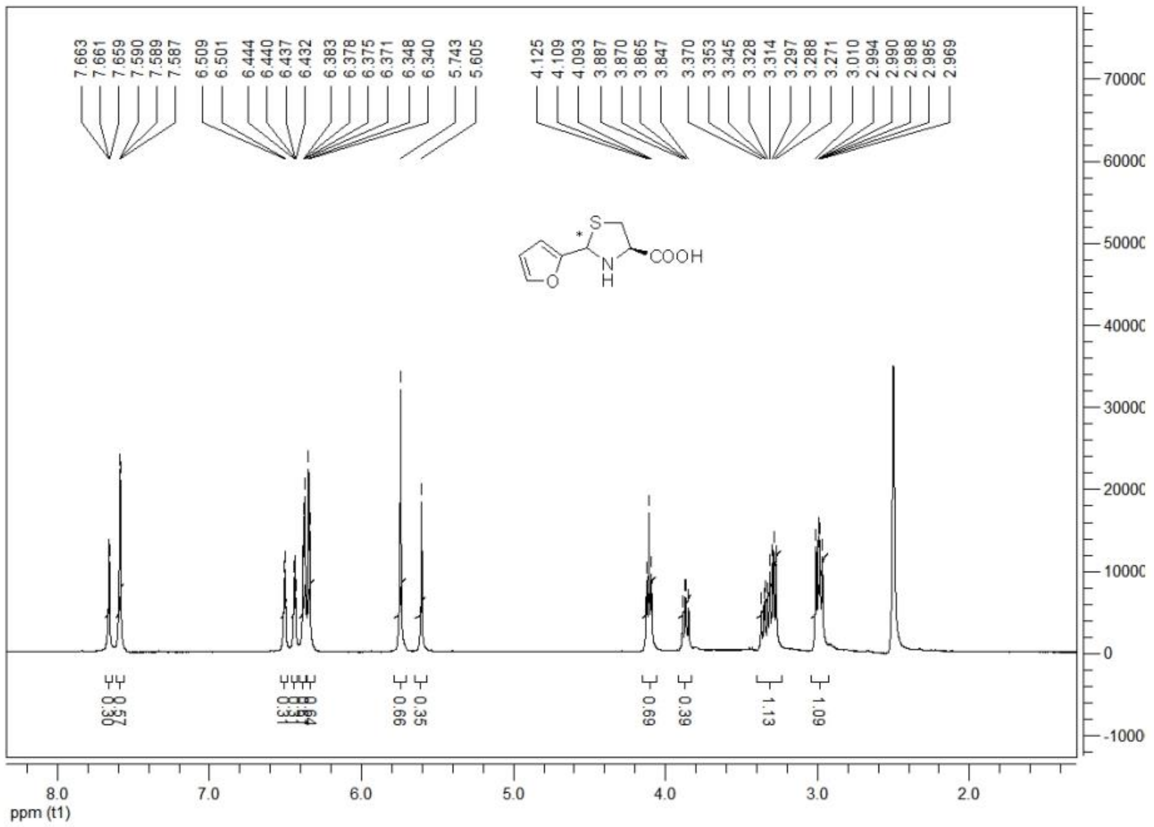
7.663
7.661
7.659
7.590
7.589
7.587
6.509
6.501
6.444
6.440
6.437
6.432
6.383
6.378
6.375
6.371
6.348
6.340
5.743
5.605
4.125
4.109
4.093
3.887
3.870
3.865
3.847
3.370
3.353
3.345
3.328
3.314
3.297
3.288
3.271
3.010
2.994
2.990
2.988
2.985
2.969
COOH
S
N
H
O
0.69
0.39
1.13
1.09
0.66
0.35
ppm (t1)

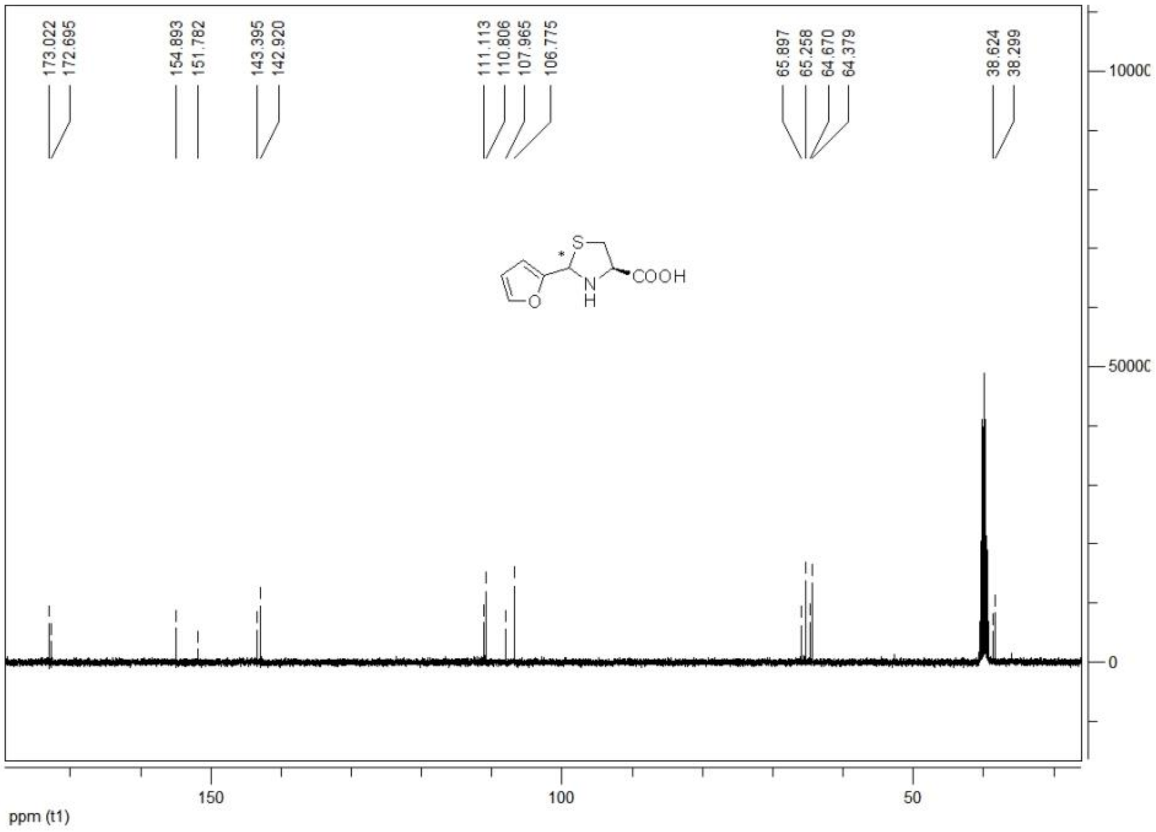
173.022
172.695
154.893
151.782
143.395
142.920
111.113
110.806
107.965
106.775
65.897
65.258
64.670
64.379
38.624
38.299
COOH
S
N
H
O
ppm (t1)

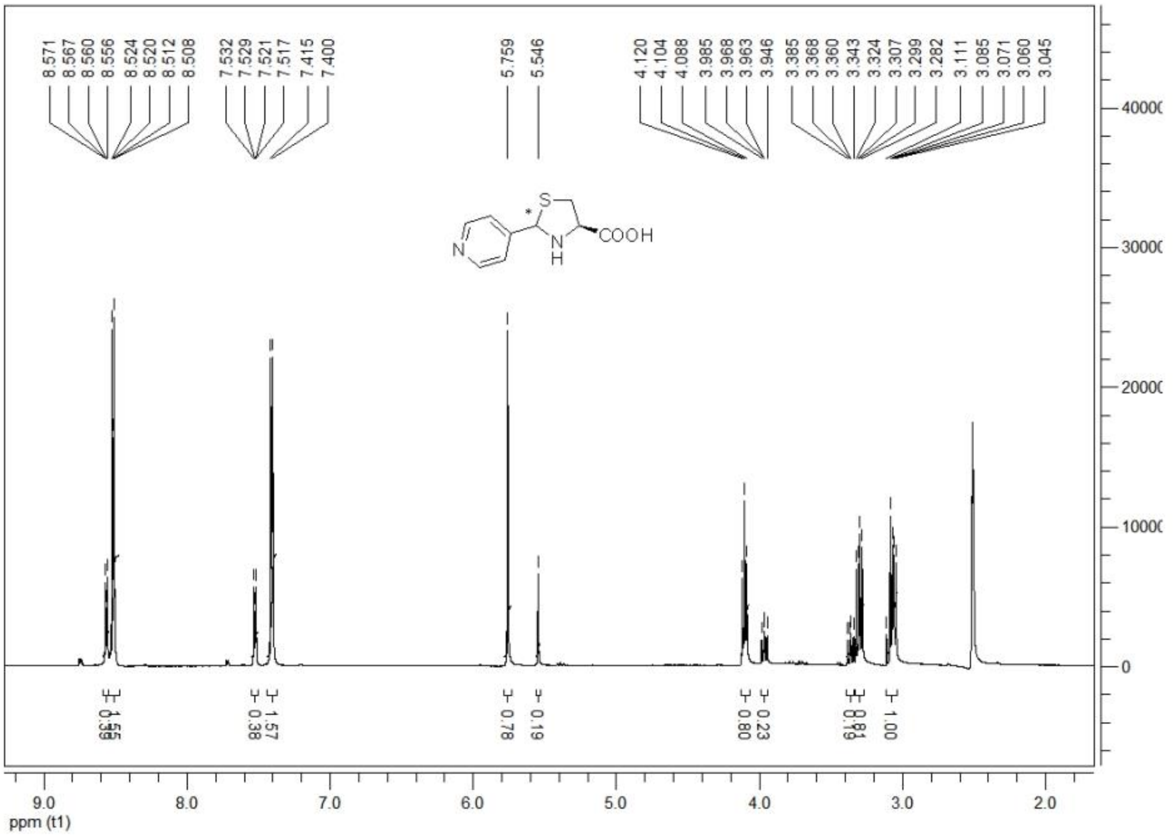
8.571
8.567
8.560
8.556
8.524
8.520
8.512
8.508
7.532
7.529
7.521
7.517
7.415
7.400
5.759
5.546
4.120
4.104
4.088
3.985
3.968
3.963
3.946
3.385
3.368
3.360
3.343
3.324
3.307
3.299
3.282
3.111
3.085
3.071
3.060
3.045
COOH
ppm (t1)

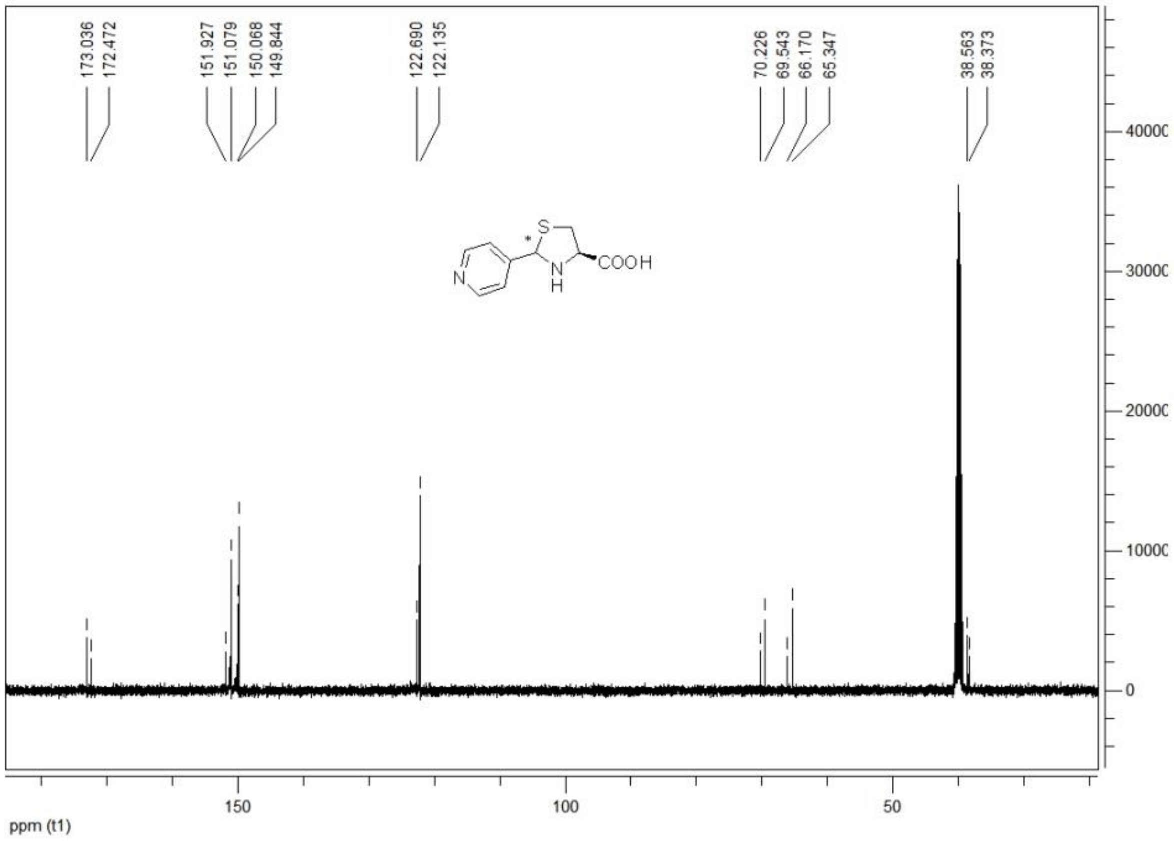
173.036
172.472
151.927
151.079
150.068
149.844
122.690
122.135
70.226
69.543
66.170
65.347
38.563
38.373
COOH
ppm (t1)

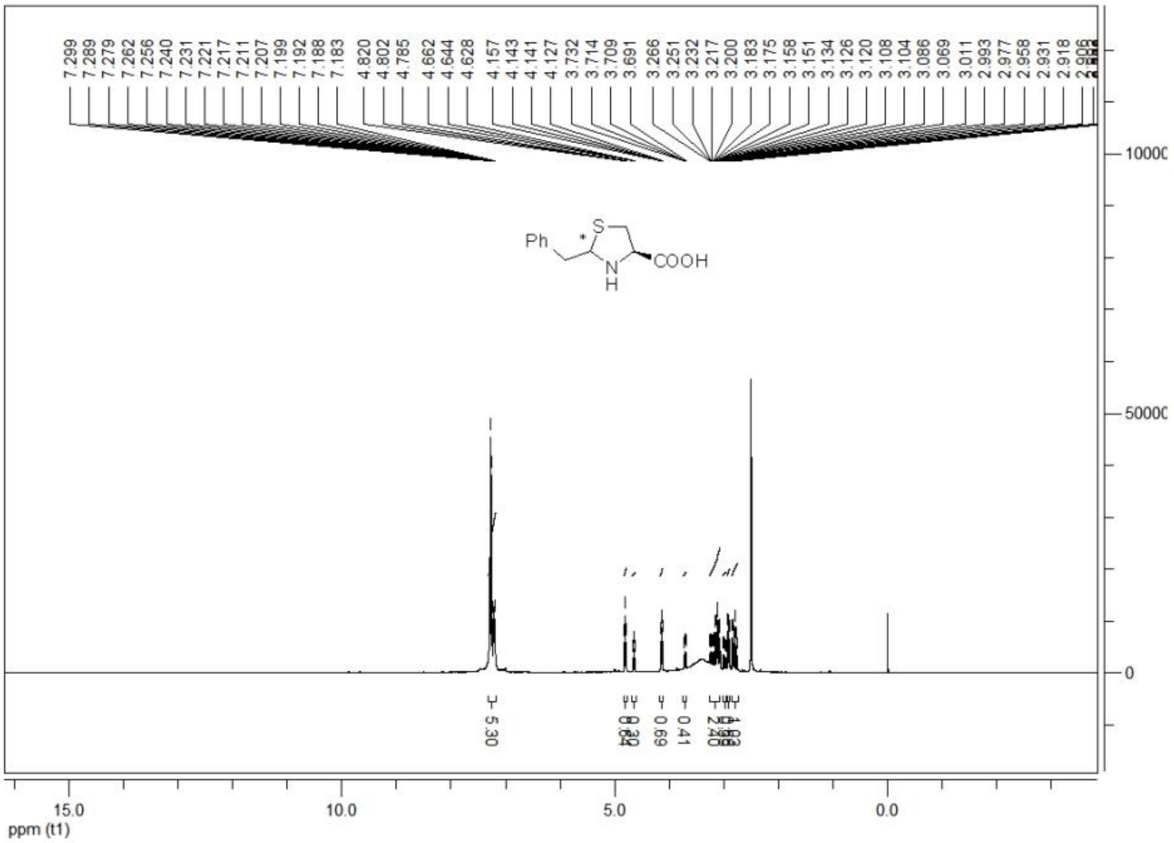
7.299
7.289
7.279
7.262
7.256
7.240
7.231
7.221
7.217
7.211
7.207
7.199
7.192
7.188
7.183
4.820
4.802
4.785
4.662
4.644
4.628
4.157
4.143
4.141
4.127
3.732
3.714
3.709
3.691
3.266
3.251
3.232
3.217
3.200
3.183
3.175
3.158
3.151
3.134
3.126
3.120
3.108
3.104
3.086
3.069
3.011
2.993
2.977
2.958
2.931
2.918
Ph
S
N
H
COOH
10000
50000
0
5.30
0.69
0.41
15.0
10.0
5.0
0.0
ppm (t1)

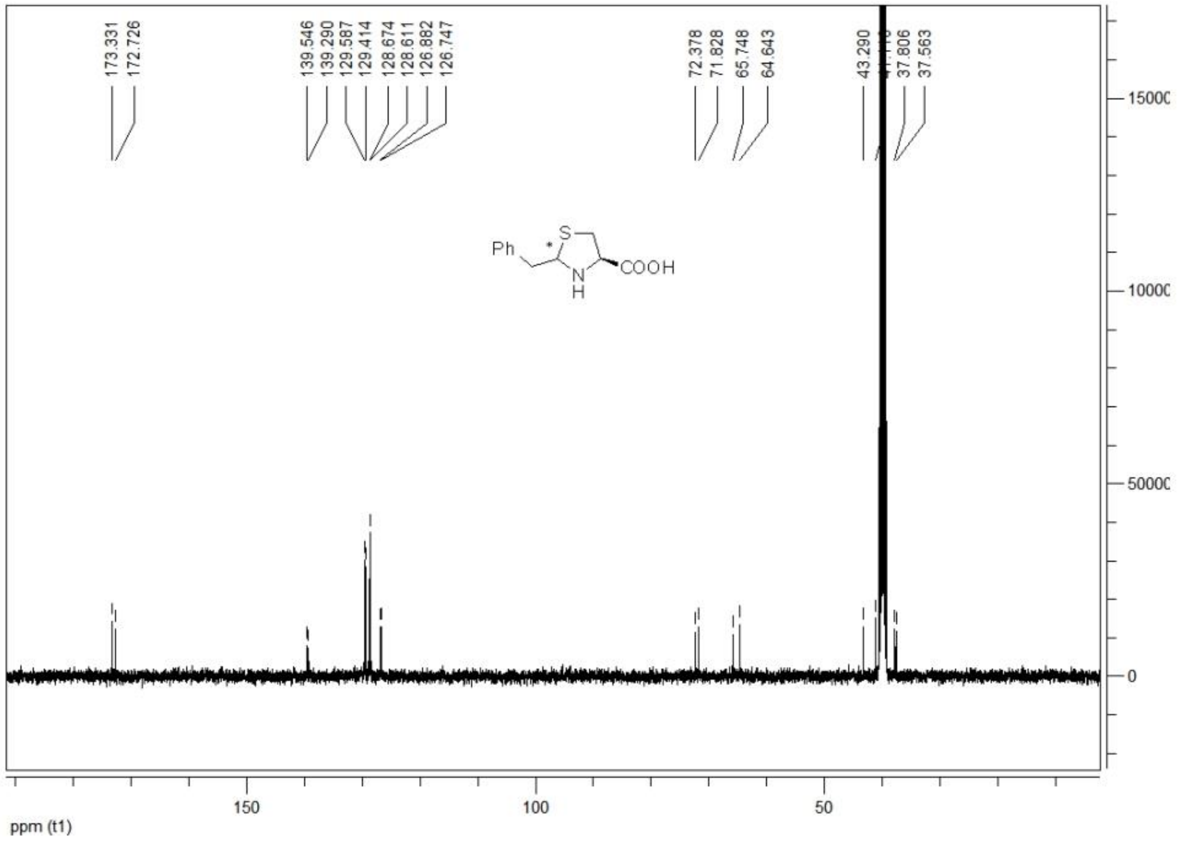
173.331
172.726
139.546
139.290
129.587
129.414
128.674
128.611
126.882
126.747
72.378
71.828
65.748
64.643
43.290
37.806
37.563
Ph
S
N
H
COOH
15000
10000
50000
0
150
100
50
ppm (t1)

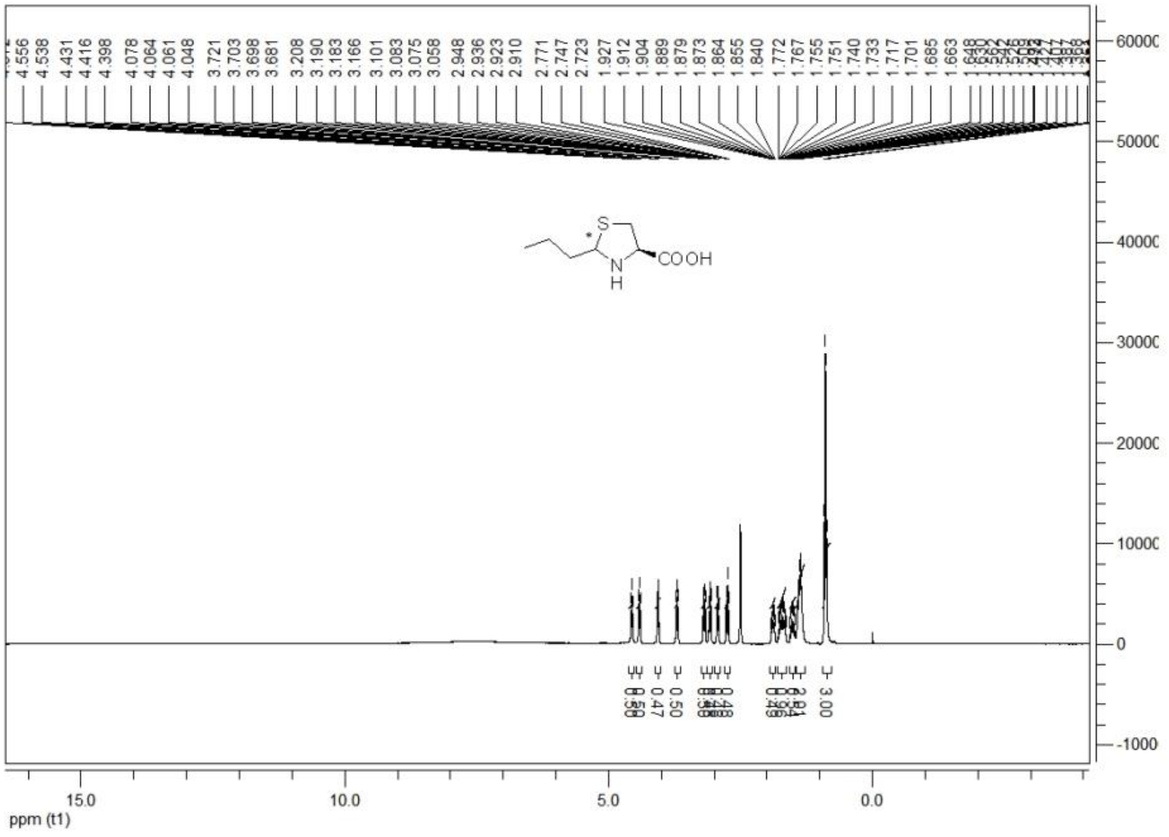
COOH
ppm (t1)

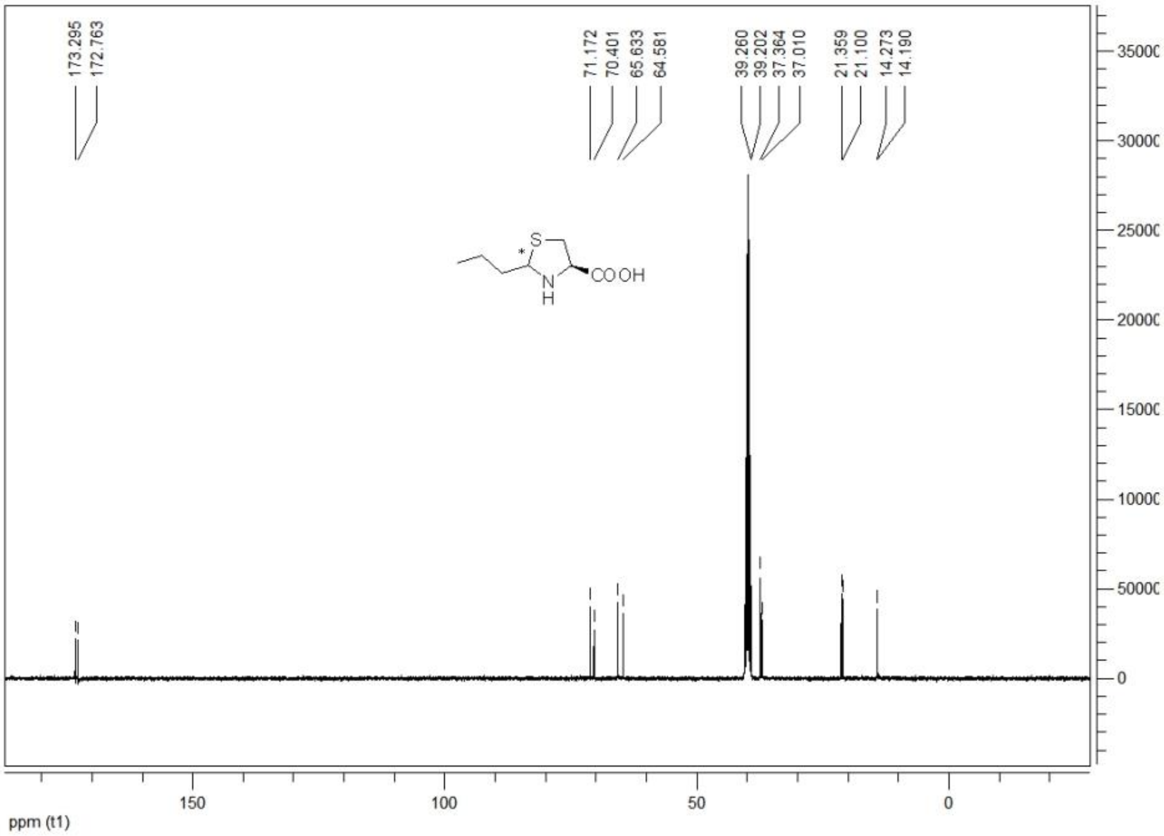
173.295
172.763
71.172
70.401
65.633
64.581
39.260
39.202
37.364
37.010
21.359
21.100
14.273
14.190
COOH
ppm (t1)

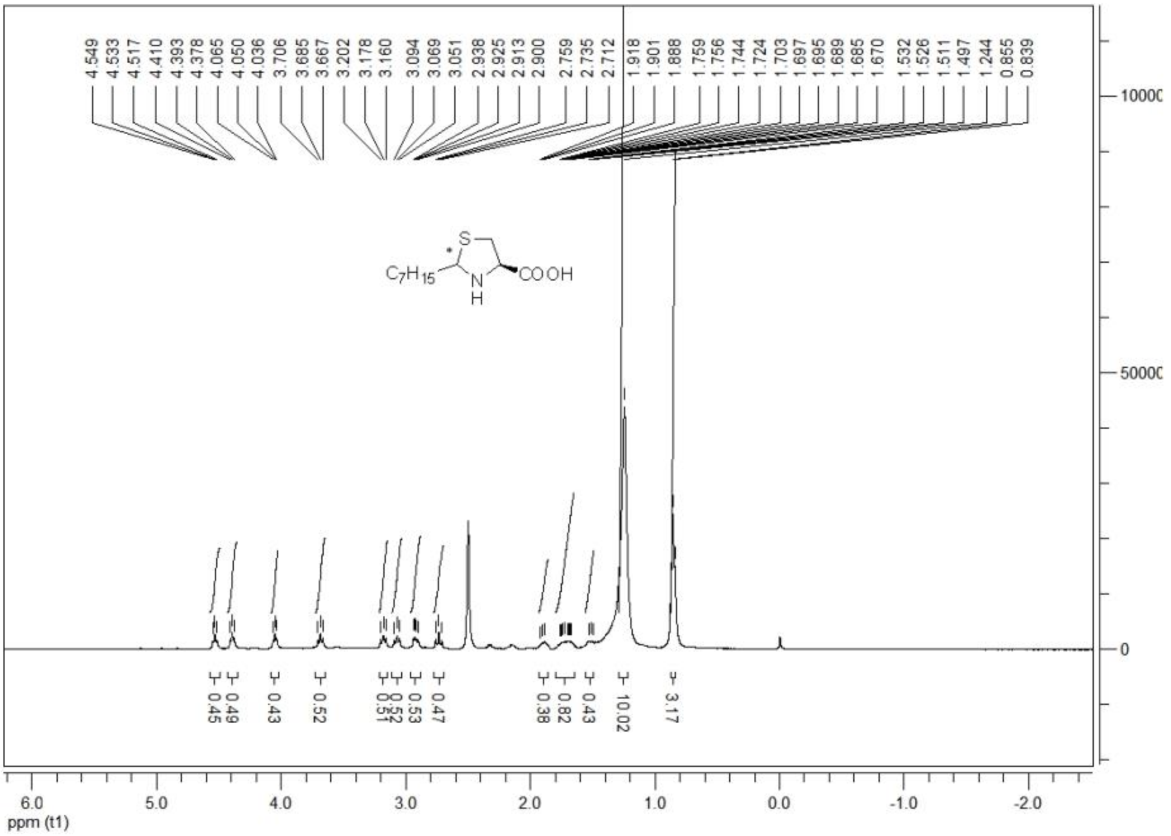
4.549
4.533
4.517
4.410
4.393
4.378
4.065
4.050
4.036
3.706
3.685
3.667
3.202
3.178
3.160
3.094
3.069
3.051
2.938
2.925
2.913
2.900
2.759
2.735
2.712
1.918
1.901
1.888
1.759
1.756
1.744
1.724
1.703
1.697
1.695
1.689
1.685
1.670
1.532
1.526
1.511
1.497
1.244
0.855
0.839
S
N
H
COOH
0.45
0.49
0.43
0.52
0.51
0.52
0.53
0.47
0.38
0.82
0.43
10.02
3.17
ppm (t1)

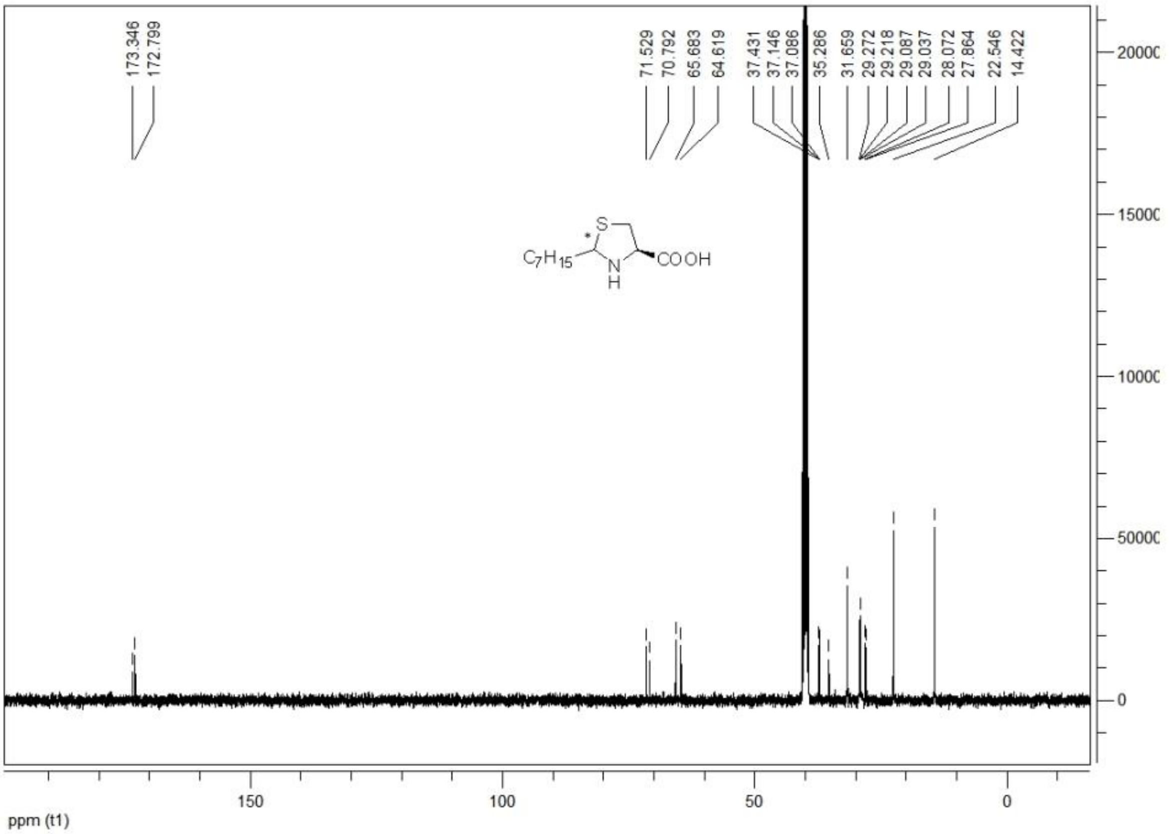
173.346
172.799
71.529
70.792
65.683
64.619
37.431
37.146
37.086
35.286
31.659
29.272
29.218
29.087
29.037
28.072
27.864
22.546
14.422
S
N
H
COOH
ppm (t1)

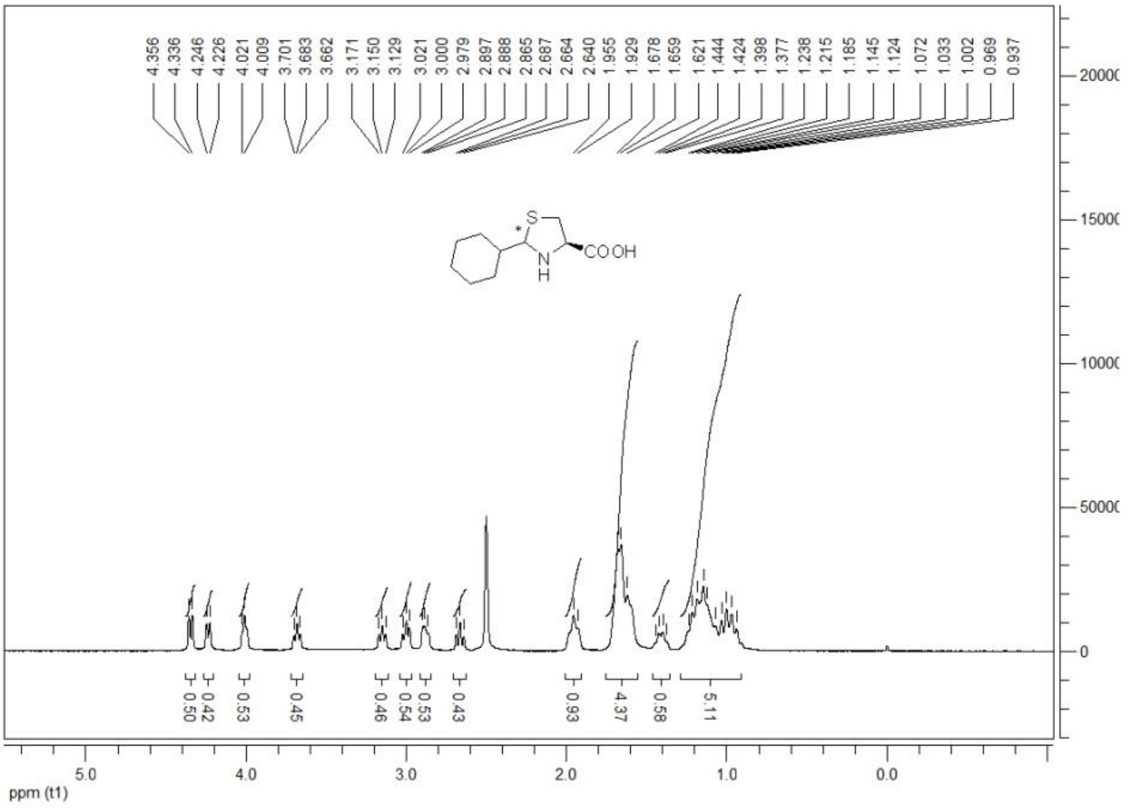

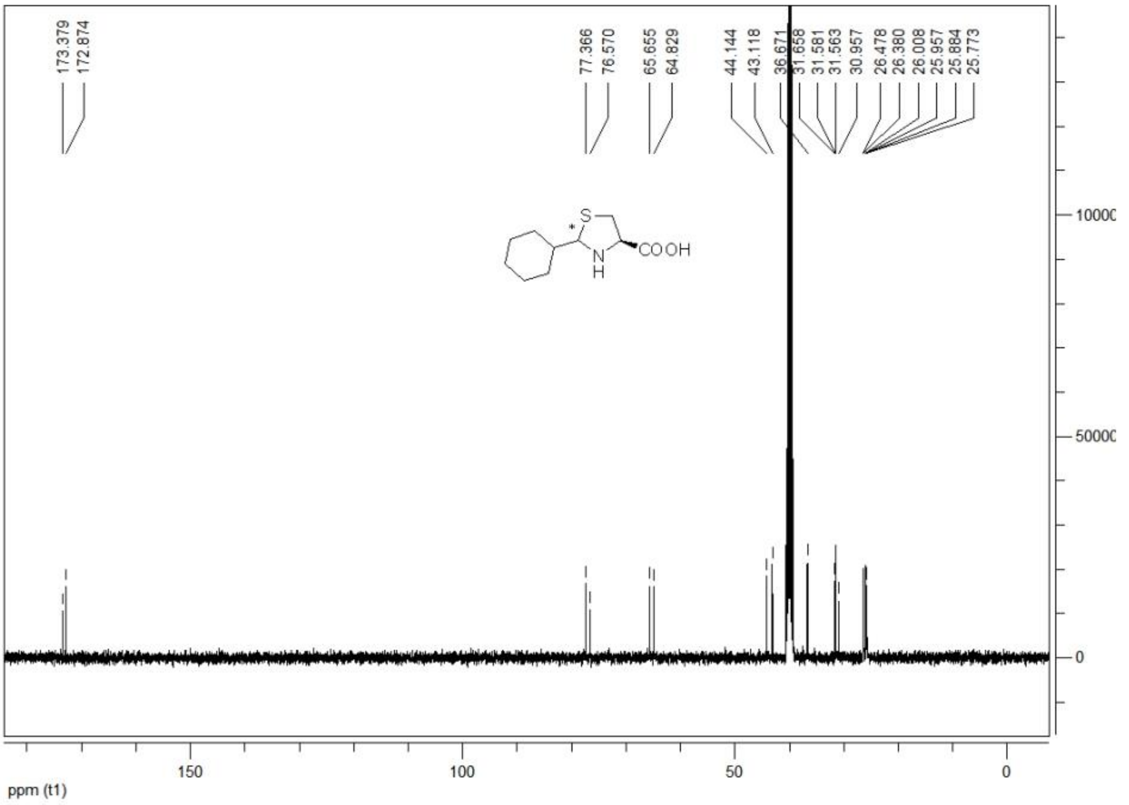

© 2020 by the authors. Submitted for possible open access publication under the terms and conditions of the Creative Commons Attribution (CC BY) license (http://creativecommons.org/licenses/by/4.0/).
